# Deficiency in astrocyte CCL2 production reduces neuroimmune control of *Toxoplasma gondii* infection

**DOI:** 10.1371/journal.ppat.1011710

**Published:** 2024-01-11

**Authors:** Stephanie B. Orchanian, Katherine Still, Tajie H. Harris, Melissa B. Lodoen

**Affiliations:** 1 Department of Molecular Biology and Biochemistry, University of California, Irvine, Irvine, California, United States of America; 2 Institute for Immunology, University of California, Irvine, Irvine, California, United States of America; 3 Center for Brain Immunology and Glia, Department of Neuroscience, University of Virginia, Charlottesville, Virginia, United States of America; University of New Mexico, UNITED STATES

## Abstract

*Toxoplasma gondii* is an obligate intracellular parasite that infects one-third of the world’s human population and establishes infection in the brain. Cerebral immune cell infiltration is critical for controlling the parasite, but little is known about the molecular cues guiding immune cells to the brain during infection. Activated astrocytes produce CCL2, a chemokine that mediates inflammatory monocyte recruitment to tissues by binding to the CCR2 receptor. We detected elevated CCL2 production in the brains of C57BL/6J mice by 15 days after *T*. *gondii* infection. Utilizing confocal microscopy and intracellular flow cytometry, we identified microglia and brain-infiltrating myeloid cells as the main producers of CCL2 during acute infection, and CCL2 was specifically produced in regions of parasite infection in the brain. In contrast, astrocytes became the dominant CCL2 producer during chronic *T*. *gondii* infection. To determine the role of astrocyte-derived CCL2 in mobilizing immune cells to the brain and controlling *T*. *gondii* infection, we generated GFAP-Cre x CCL2^fl/fl^ mice, in which astrocytes are deficient in CCL2 production. We observed significantly decreased immune cell recruitment and increased parasite burden in the brain during chronic, but not acute, infection of mice deficient in astrocyte CCL2 production, without an effect on peripheral immune responses. To investigate potential mechanisms explaining the reduced control of *T*. *gondii* infection, we analyzed key antimicrobial and immune players in host defense against *T*. *gondii* and detected a reduction in iNOS^+^ myeloid cells, and *T*. *gondii*-specific CD4^+^ T cells in the knockout mice. These data uncover a critical role for astrocyte-derived CCL2 in immune cell recruitment and parasite control in the brain during chronic, but not acute, *T*. *gondii* infection.

## Author summary

Neuroinflammation plays an important role in protecting the brain against infection, but it must be controlled to prevent damage to the brain. *Toxoplasma gondii* is an obligate intracellular foodborne parasite that infects one-third of the world’s human population. Among pathogens, *T*. *gondii* has the rare ability to cross the blood-brain barrier and establish chronic infection in the brain, inducing a protective, but non-sterilizing immune response. In this study, we examined *T*. *gondii* infection of mice to understand the factors driving the neuroinflammatory response to infection of the brain. We determined that the cells producing the potent chemokine, CCL2, changed over the course of infection: myeloid cells were the main CCL2 producers during acute infection, whereas astrocytes became the dominant CCL2 producers during chronic infection. Additionally, the ablation of CCL2 production specifically from astrocytes reduced immune cell recruitment to the brain and decreased control of the parasite during chronic infection. Therefore, astrocyte-derived CCL2 plays a critical role in the control of chronic, but not acute, *T*. *gondii* infection.

## Introduction

An effective neuroinflammatory response against infection requires the accuracy and precision to enable sufficient immune cell infiltration to the brain to control the pathogen while preserving the brain itself. The recruitment of peripheral immune cells to the brain is a highly orchestrated process driven in part by the brain’s chemokine milieu. The unique repertoire of chemokine receptors expressed on circulating immune cells allows them to be recruited by distinct combinations of chemokines. In addition, as cells infiltrate the brain, their own chemokine production can further alter the landscape, drawing other immune cells into the tissue. Therefore, the evolving chemokine environment in the brain enables targeted and precisely timed immune cell mobilization to protect against infections of the brain.

*Toxoplasma gondii* is an obligate intracellular parasite with the unique ability to traverse the blood-brain barrier and infect the brain [1,2]. *T*. *gondii* is among the most successful parasites, as it can invade almost all nucleated cells, infect virtually all warm-blooded animals, and is estimated to have infected one-third of the global human population [3,4]. This parasite is transmitted via ingestion when humans consume water contaminated with oocysts or undercooked meat containing tissue cysts, or can spread vertically via congenital infection [5–8]. After ingestion, *T*. *gondii* is able to cross the intestinal epithelium, enter the bloodstream, and disseminate throughout the body, eventually reaching the brain where it establishes a chronic infection [9].

*T*. *gondii* has a lytic life cycle, and the rapidly replicating tachyzoite stage during acute infection can cause cellular damage as the parasites lyse out of infected cells and invade neighboring host cells [10]. In most immunocompetent individuals, infection is asymptomatic, but *T*. *gondii* stage converts into cyst-forming bradyzoites, which are slow-growing and found predominantly in neurons and muscle cells during chronic infection [11,12]. However, in immunocompromised people, *T*. *gondii* can be potentially fatal due to loss of effective immune control [13], and cerebral toxoplasmosis causes 10% of AIDS-related deaths in Africa [14].

During the initial infection, immune cells, including dendritic cells (DCs), monocytes, and macrophages recognize *T*. *gondii’s* glycosylphosphatidylinositol anchor and profilin, leading to the production of IL-12 via TLR2, 4, 11, and 12 [15–19]. TLR11 and 12 recognition of *T*. *gondii* profilin is specific to mice, as these TLRs are not functional in humans. IL-12 production by DCs activates NK cells and T_H_1 cells to produce IFN-γ [20–22]. IFN-γ plays an important role in controlling *T*. *gondii* infection, including upregulating the expression of inducible nitric oxide synthase, indoleamine 2,3-dioxygenase, guanylate binding proteins, and immunity-related GTPases, all of which aid the infected host cell in killing *T*. *gondii* [23–26].

Inflammatory monocytes play a critical role in immune defense against the parasite during both the acute and chronic stages of the infection [27–29]. These cells express high levels of the chemokine receptor CCR2, which binds to its ligand, the chemoattractant CCL2 [30]. CCR2^+^ monocyte infiltration of the brain coincides with parasite entry to the brain during acute infection, and these cells persist in the brain during the chronic stage of infection [31]. However, the factors driving this recruitment are poorly understood.

The production of CCL2 during *T*. *gondii* infection is necessary for survival, as mice lacking this chemokine have decreased monocyte recruitment and succumb to acute *T*. *gondii* infection [28]. During infection, CCL2 recruits CCR2^+^ monocytes out of the bone marrow into the circulation, and ultimately into injured or inflamed tissues [32]. In the periphery in mice, CCL2 is produced during recognition of the *T*. *gondii* protein profilin, which leads to the recruitment of Ly6C^hi^ monocytes to sites of infection [33]. *In vitro*, CCL2 production is triggered by the release of the alarmin S100A11 by *T*. *gondii*-infected myeloid cells [34].

In the brain, glial cells can produce CCL2 in the context of neuroinflammatory diseases [35,36], and during chronic *T*. *gondii* infection, astrocytes comprise 75% of the CCL2-producing cells [37]. Additionally, if astrocyte activation is suppressed, the levels of CCL2 and of CCR2^+^ cells decrease in the brain [38]. We aimed to determine the role of astrocyte-derived CCL2 in immune cell recruitment and control of *T*. *gondii* from acute to chronic infection of the mouse brain. We found that microglia and brain-infiltrating myeloid cells produce high levels of CCL2 during acute infection in close proximity to the parasites. In contrast, astrocytes become the dominant CCL2 producers during chronic *T*. *gondii* infection. By infecting mice specifically deficient in astrocyte CCL2 production (GFAP-Cre x CCL2^fl/fl^), we observed significantly decreased immune cell recruitment to the brain and reduced parasite control during chronic, but not acute, infection.

## Results

### CCL2 is produced by astrocytes, microglia, and infiltrating myeloid cells during acute *T*. *gondii* infection

**Fig 1:**
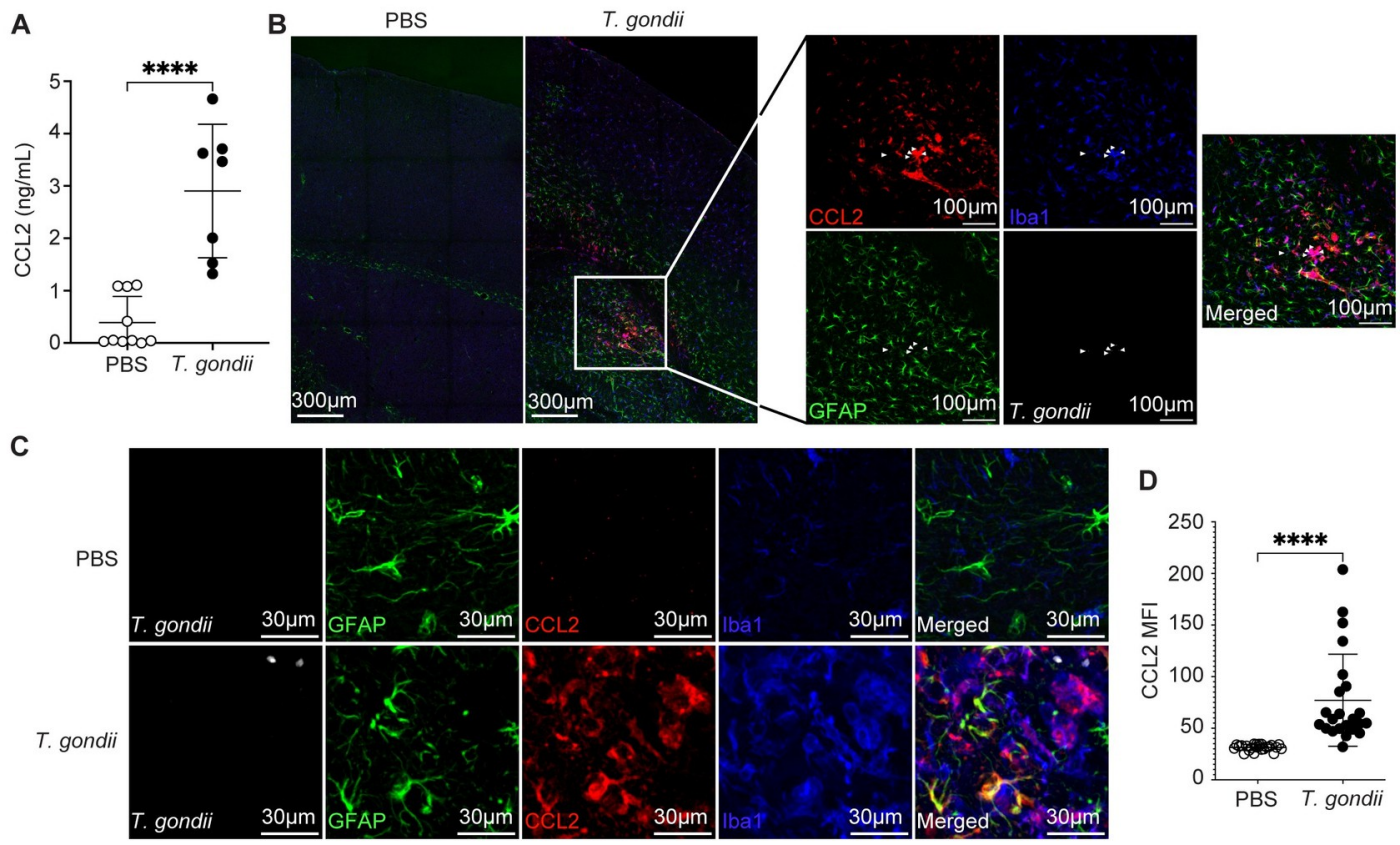
CCL2 production increases in the brain during acute *Toxoplasma gondii* infection. C57BL/6 (A) or CCL2-RFP mice (B-D) were injected with PBS or infected with type II *Prugniaud* (PRU) strain *T*. *gondii*, and brains were harvested and examined at 15 DPI. (A) CCL2 protein levels were measured in whole brain homogenates by ELISA. *n* = 7 to 10 mice per group from 4 independent experiments. (B) Representative tile scan of *T*. *gondii* (white), GFAP^+^ astrocytes (green), CCL2-RFP (red), and Iba1^+^ myeloid cells (blue) in brains of infected or PBS control mice. Magnified inset shows a FOV with *T*. *gondii* parasites (white arrowheads). (C) Representative confocal microscopy of *T*. *gondii* (white), GFAP^+^ astrocytes (green), CCL2-RFP (red), and Iba1^+^ myeloid cells (blue) in brains of infected or PBS control mice. (D) Quantification of mean fluorescence intensity (MFI) of CCL2-RFP in FOVs containing CCL2 in infected brains, and in the same brain regions in PBS control mice. *n* = 23–24 FOV from 6 mice per group from 3–4 independent experiments. Statistical significance was determined by Student’s *t*-test (A) and (D). ****p<0.0001.

CCL2 is transcribed in the brain as early as 7 days post-infection (DPI) and persists at 10, 30, and 60 DPI [39]. To examine CCL2 protein levels in the brain, wild-type C57BL/6J mice were injected intraperitoneally (i.p.) with 200 type II *T*. *gondii* tachyzoites or PBS as a control. The brains were harvested at 15 DPI, which is an acute infection timepoint that represents the peak of CCR2^+^ monocyte infiltration to the brain in our model [31]. Brains were snap frozen and homogenized, and CCL2 levels in brain homogenates were analyzed by ELISA. We detected a significant increase in CCL2 protein levels by 15 DPI (Fig 1A). To investigate the cells that produce CCL2 during acute infection, we used CCL2-RFP reporter mice, in which all cells expressing CCL2 also express RFP [40]. The CCL2-RFP mice were injected with PBS or infected with *T*. *gondii* as above, and the brains were harvested at 15 DPI and sectioned for confocal microscopy. We stained for GFAP^+^ astrocytes, CCL2-producing (RFP^+^) cells, and Iba-1^+^ cells, which include microglia, macrophages, and mature monocytes, as these cells have been found to produce CCL2 in other neuroinflammatory diseases, including multiple sclerosis [41]. We found significantly more GFAP^+^, Iba-1^+^, and CCL2-RFP^+^ cells in the brains of *T*. *gondii*-infected mice compared to those of PBS-injected mice (Fig 1B). Most of the CCL2^+^ cells were Iba-1^+^ myeloid cells, with some CCL2^+^ astrocytes at 15 DPI (Fig 1C). The mean fluorescence intensity (MFI) of CCL2-RFP was significantly higher in *T*. *gondii-*infected mice than in the same regions of the brains of control PBS-injected mice (Fig 1D).

**Fig 2:**
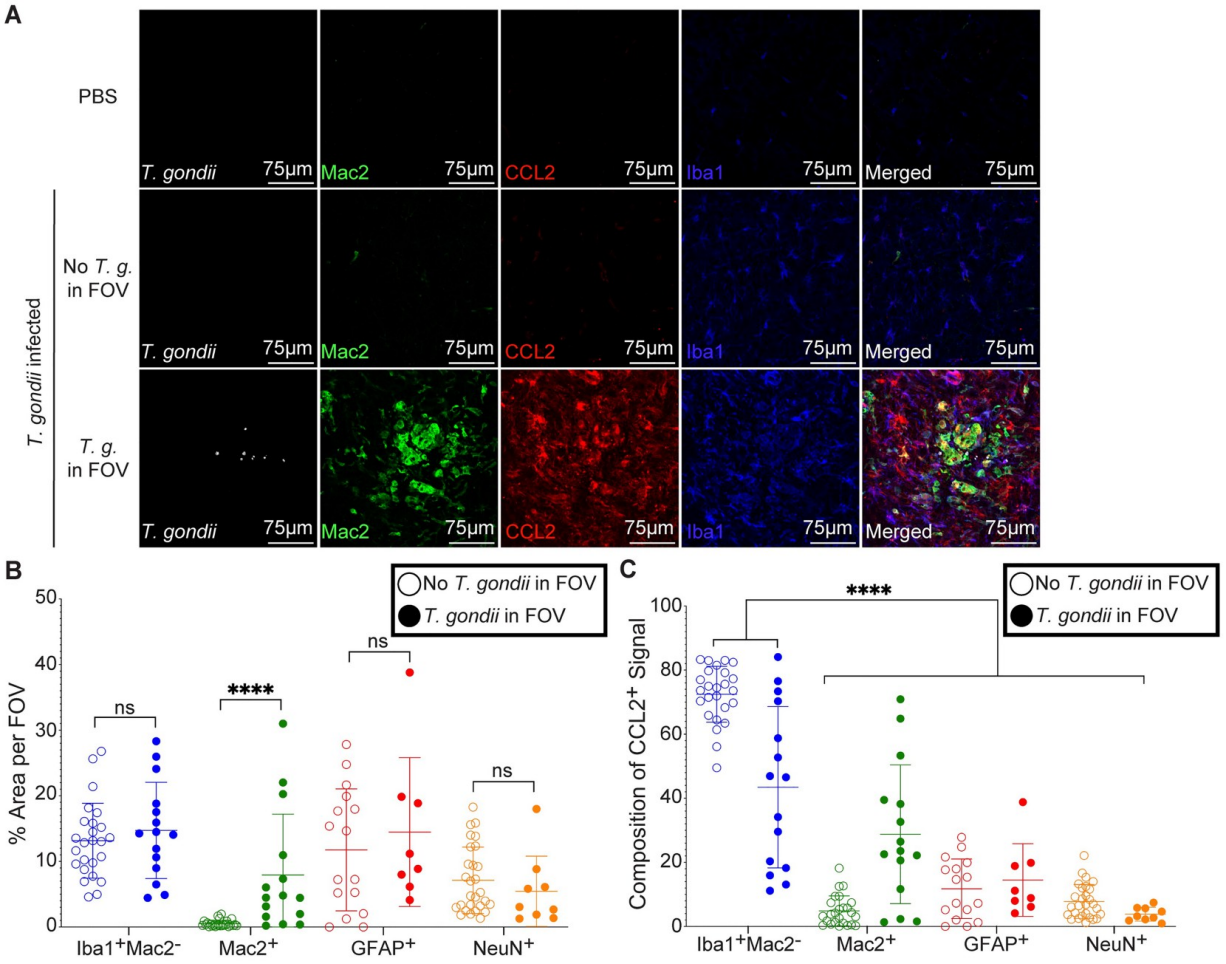
Production of CCL2 by cell type during acute *T*. *gondii* infection. CCL2-RFP mice were injected with PBS or infected with PRU strain *T*. *gondii*, and brains were harvested at 15 DPI. (A) Representative confocal microscopy of *T*. *gondii* (white), Mac2^+^ myeloid cells (green), CCL2-RFP (red), and Iba1^+^ myeloid cells (blue) in brains of PBS control mice or *T*. *gondii-*infected mice in FOV with or without parasites. (B) Percent area of each cell type within FOV with or without parasites from infected mice. (C) Percent area of each cell type within CCL2^+^ area in FOV with or without parasites from infected mice. *n* = 8–25 FOV from 4–5 mice per group from 4–5 independent experiments. Statistical significance was determined by two-way ANOVA. ****p<0.0001, ns: not significant.

To further analyze the Iba-1^+^ CCL2-producing cells we stained brain sections for both Iba-1 and Mac2, which predominantly stains infiltrating myeloid cells [42], but is also expressed in some microglia [43]. In the brains of infected mice, we observed a significant increase in Mac2^+^ cells specifically in FOV containing parasites (Fig 2A and 2B, green circles). By focusing on the CCL2-RFP^+^ cells, we detected most of the CCL2-RFP signal within Iba-1^+^Mac2^-^ microglia (Fig 2C, blue circles). Interestingly, CCL2-RFP signal was also detected within Mac2^+^ positive cells, suggesting that infiltrating myeloid cells that are recruited to sites of *T*. *gondii* infection are also producing CCL2 (Fig 2C, green circles). To further characterize the CCL2-RFP^+^ myeloid cells, we utilized an anti-Ly6C antibody, which stains monocytes, and found CCL2-RFP signal within Ly6C^+^ cells (S1A Fig). We confirmed these findings by staining with anti-Ly6B.2, which stains both infiltrating neutrophils and monocytes, but not lymphocytes, and also observed colocalization of CCL2-RFP and Ly6B.2 (S1B Fig). In addition to infiltrating myeloid cells, microglia, and astrocytes, it has been shown that neurons can produce CCL2 in a model of viral CNS infection [44]. To examine the relative contribution of neurons to CCL2 signal in the brain, we also stained brain sections with antibodies against the neuronal marker NeuN and compared CCL2 signal in NeuN^+^ neurons to that of Iba-1^+^Mac2^-^ microglia, Mac2^+^ myeloid cells, and GFAP^+^ astrocytes in FOVs with and without parasites. Although neurons were readily detectable in these FOVs (Figs 2B and S1C), there was little to no CCL2 signal within these cells (Figs 2C and S1C), indicating that neurons are not likely to be a substantial source of CCL2 in the *T*. *gondii-*infected brain at this acute timepoint.

**Fig 3:**
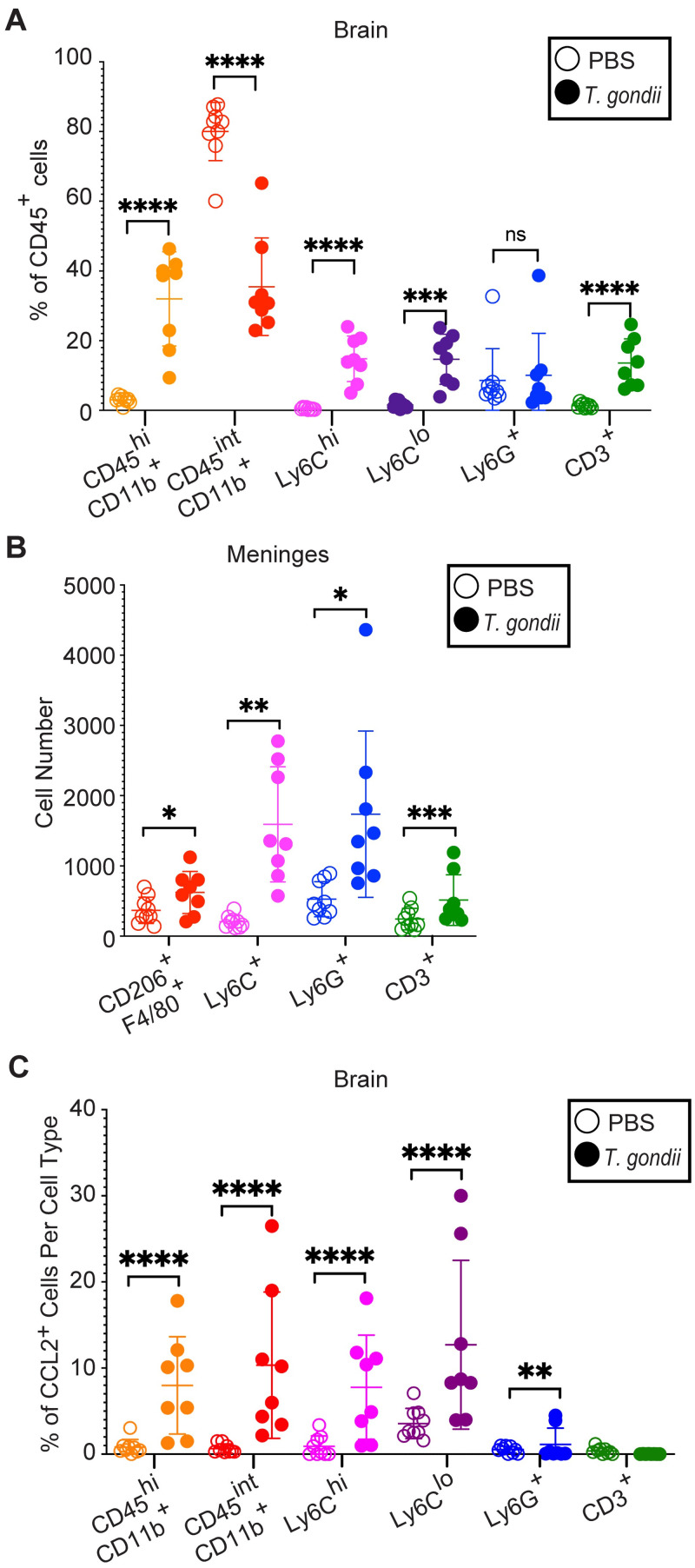
CCL2 production by myeloid cells in the brain during acute *T*. *gondii* infection. CCL2-RFP mice were injected with PBS as a control or infected with PRU strain *T*. *gondii* and examined at 15 DPI. (A) Percentage of immune cells out of CD45^+^ cells in the brains of PBS-injected (open circles) or *T*. *gondii*-infected (closed circles) mice. Cells were identified as infiltrating myeloid cells (CD45^hi^CD11b^+^), microglia (CD45^int^ CD11b^+^), inflammatory monocytes (CD45^+^CD11b^+^Ly6C^hi^), patrolling monocytes (CD45^+^CD11b^+^Ly6C^lo^), neutrophils (CD45^+^Ly6G^+^), or T cells (CD45^+^CD3^+^). (B) Immune cell numbers in the meninges of PBS-injected (open circles) or *T*. *gondii*-infected (closed circles) mice. Cell types were identified as in (A) with the addition of meningeal macrophages (CD45^+^CD11b^+^CD206^+^F4/80^+^). (C) Frequencies of CCL2^+^ cells within each immune cell population in the brains of PBS-injected (open circles) or *T*. *gondii*-infected (closed circles) mice. *n* = 8–9 mice per group from three independent experiments. Statistical significance was determined by a randomized block ANOVA. *p<0.05, **p<0.01, ***p<0.001, ****p<0.0001, ns: not significant.

To quantify immune cell mobilization to the brain and meninges, we utilized flow cytometry with a sequential gating strategy (S2A Fig). As previously reported [31], we detected an increase in the frequencies of infiltrating myeloid cells (CD45^hi^ CD11b^+^), including inflammatory monocytes (CD45^+^ CD11b^+^Ly6C^hi^), patrolling monocytes (CD45^+^ CD11b^+^Ly6C^lo^), and T cells (CD45^+^ CD3^+^) from among total CD45^+^ immune cells in the brains of infected compared to PBS-injected control mice (Fig 3A). Since the meninges is separated from the brain by the thin layer of astrocyte processes known as the glia limitans [45], we examined whether meningeal inflammation mirrored that of the brain [42]. We found that *T*. *gondii* infection increased the numbers of macrophages (CD45^+^ F4/80^+^CD206^+^), monocytes (CD45^+^Ly6C^+^), neutrophils (CD45^+^Ly6G^+^), and T cells (CD45^+^CD3^+^) in the meninges at 15 DPI (Figs 3B and S2D).

We also used flow cytometry to determine the frequencies of immune cells producing CCL2 in the brains of control or *T*. *gondii-*infected mice at 15 DPI. We found that the percent of microglia (CD45^int^ CD11b^+^), infiltrating myeloid cells (CD45^hi^CD11b^+^), including Ly6C^hi^ and Ly6C^lo^ monocytes, and neutrophils (Ly6G^+^) producing CCL2-RFP increased in infected compared to control mice (Fig 3C), consistent with the microscopy data (Fig 2B). On average, 10.33% of microglia, 7.77% of Ly6C^hi^ and 12.71% of Ly6C^lo^ monocytes, and 1.14% of neutrophils were also positive for CCL2-RFP during infection (Fig 3C). We also examined the mean fluorescence intensity (MFI) of the CCL2-RFP within each cell population in control and infected mice and observed increases in the CCL2-RFP MFI of infiltrating myeloid cells, microglia, and Ly6C^lo^ monocytes from infected mice (S3 Fig). Therefore, during acute infection, immune cells are recruited to the brain and meninges. Although resident microglia comprise the majority of CCL2-producing cells in the brain, Ly6C^hi^ and Ly6C^lo^ monocytes, neutrophils, and astrocytes also produce CCL2 at this timepoint.

### Astrocyte-derived CCL2 is not required for immune cell recruitment and control of acute *T*. *gondii* infection

**Fig 4:**
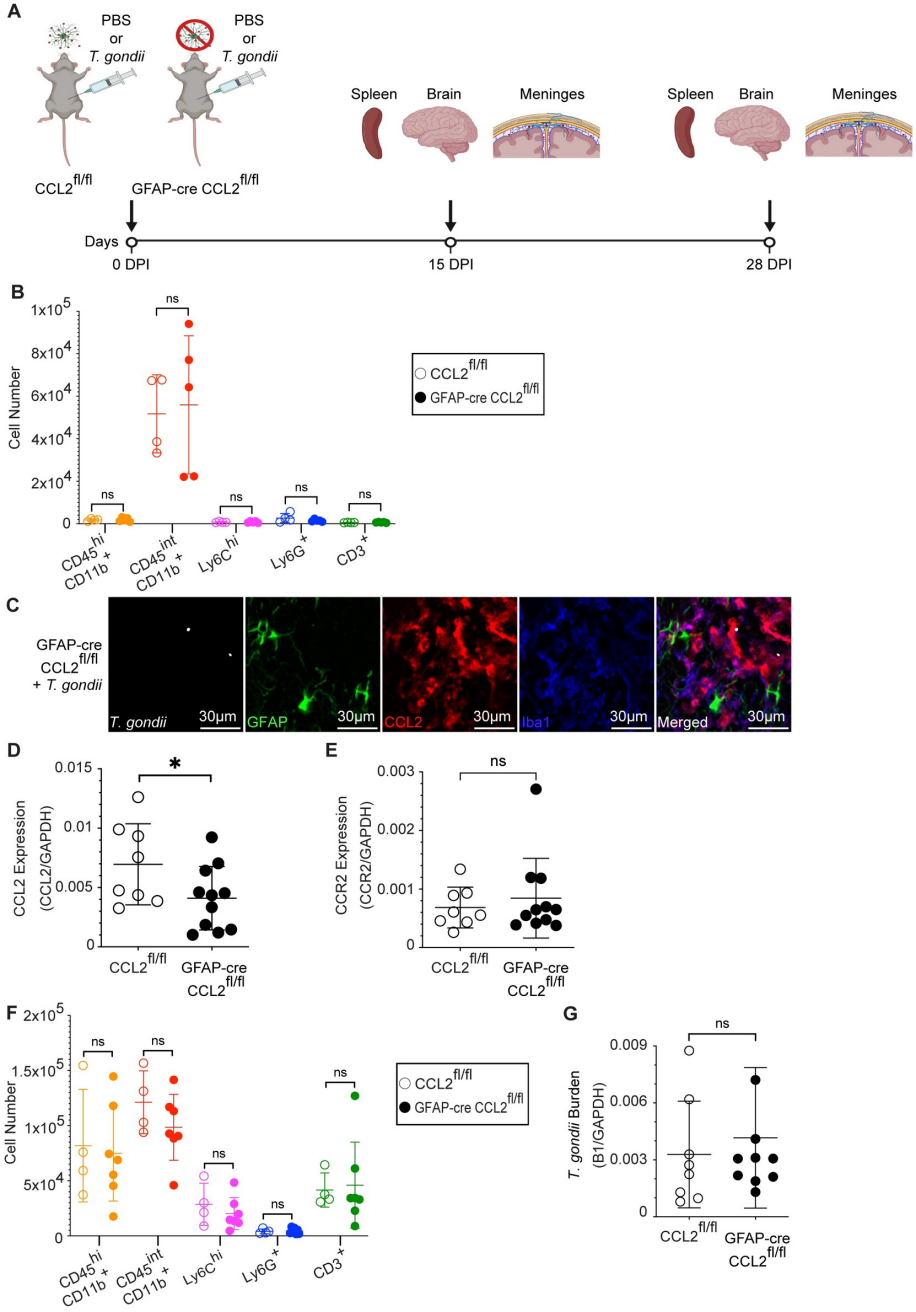
Immune cell recruitment to the brain and parasite burden during acute *T*. *gondii* infection of mice deficient in astrocyte-derived CCL2. (A) Experimental design, created with Biorender. (B) CCL2^fll/fl^ (open circles) and GFAP-cre CCL2^fl/fl^ (closed circles) mice were injected with PBS, and immune cell numbers in the brain were quantified by flow cytometry 15 days later. (C) Representative confocal microscopy of *T*. *gondii* (white), GFAP^+^ astrocytes (green), CCL2-RFP (red), and Iba1^+^ myeloid cells (blue) in GFAP-cre CCL2^fl/fl^ mice at 15 DPI with PRU strain *T*. *gondii*. (D and E) qPCR for *ccl2* (D) or *ccr2* (E) transcripts from brains of CCL2^fl/fl^ and GFAP-cre CCL2^fl/fl^ mice at 15 DPI. Transcripts are normalized to *gapdh*. (F) Quantification of brain immune cells by flow cytometry from CCL2^fl/fl^ and GFAP-cre CCL2^fl/fl^ mice at 15 DPI. (G) Assessment of brain parasite burden by qPCR for B1 from CCL2^fl/fl^ and GFAP-cre CCL2^fl/fl^ mice at 15 DPI. In (B) *n* = 4–5 mice per group, in (D and E) *n* = 8–11 mice per group, in (F) *n* = 4–7 mice per group, and in (G) *n* = 8–9 mice per group from two to three independent experiments. Statistical significance was determined by randomized block ANOVA, *p<0.05, ns: not significant.

To elucidate the role of astrocyte-derived CCL2 during infection, we generated mice in which astrocytes were specifically deficient in CCL2 production. We bred CCL2^fl/fl^ mice to mice expressing Cre recombinase driven by the astrocyte-specific GFAP promoter (GFAP-Cre 77.6 mice [46]) to generate GFAP-Cre CCL2^fl/fl^ mice. The genotype of these mice was confirmed using PCR on genomic DNA (S4A and S4B Fig). We first ensured that the knockout of astrocyte-derived CCL2 did not affect immune cell frequencies in the brain or periphery in the absence of infection. GFAP-Cre CCL2^fl/fl^ and control CCL2^fl/fl^ mice were intraperitoneally injected with PBS (Fig 4A), the mice were euthanized, and their spleens, brains, and meninges were harvested 15 days later. We detected no differences in the myeloid or lymphoid immune cell subsets in the spleen (S5A Fig), the meninges (S5B Fig), nor the brain (Fig 4B).

To determine the role of astrocyte-derived CCL2 in early CCR2^+^ cell recruitment and parasite control, we infected GFAP-Cre CCL2^fl/fl^ and control CCL2^fl/fl^ mice as above with 200 type II GFP-expressing *T*. *gondii* and collected the spleen, meninges, and brain at 15 DPI (Fig 4A). By conducting confocal microscopy on brain sections from infected GFAP-Cre CCL2^fl/fl^ mice, we confirmed that GFAP^+^ astrocytes did not express CCL2-RFP, indicating knockout of astrocyte-derived CCL2 in infected mice (Fig 4C). We also detected a decrease in *ccl2* transcripts in the brain in GFAP-Cre CCL2^fl/fl^ during acute infection (Fig 4D). In contrast, *ccr2* transcripts in the brain were similar in control CCL2^fl/fl^ mice and GFAP-Cre CCL2^fl/fl^ mice during infection (Fig 4E). We also examined which cells expressed CCR2 in the *T*. *gondii-*infected brain and found that the vast majority (>90% on average) of CCR2-RFP-expressing cells were monocytes (S6 Fig). To determine if any specific immune cell subsets were affected by the loss of astrocyte-derived CCL2, we conducted flow cytometry on brain homogenates during infection and found no differences in immune cell recruitment to the brain (Fig 4F). There were also no differences in immune cell frequencies in the spleen or meninges of mice deficient in astrocyte-derived CCL2 (S5C and S5D Fig). Finally, to determine if the loss of astrocyte-derived CCL2 affected the ability of the mice to control *T*. *gondii* infection, we measured levels of the parasite B1 gene in the brain by qPCR and detected no difference in B1 levels in the GFAP-Cre CCL2^fl/fl^ mice compared to CCL2^fl/fl^ mice at 15 DPI (Fig 4G). With these results, we concluded that astrocyte-derived CCL2 does not affect peripheral immune cell mobilization to the brain or meninges, nor control of parasite burden at this timepoint.

### Astrocyte-derived CCL2 induces immune cell recruitment to the brain to control parasite burden during chronic *T*. *gondii* infection

**Fig 5:**
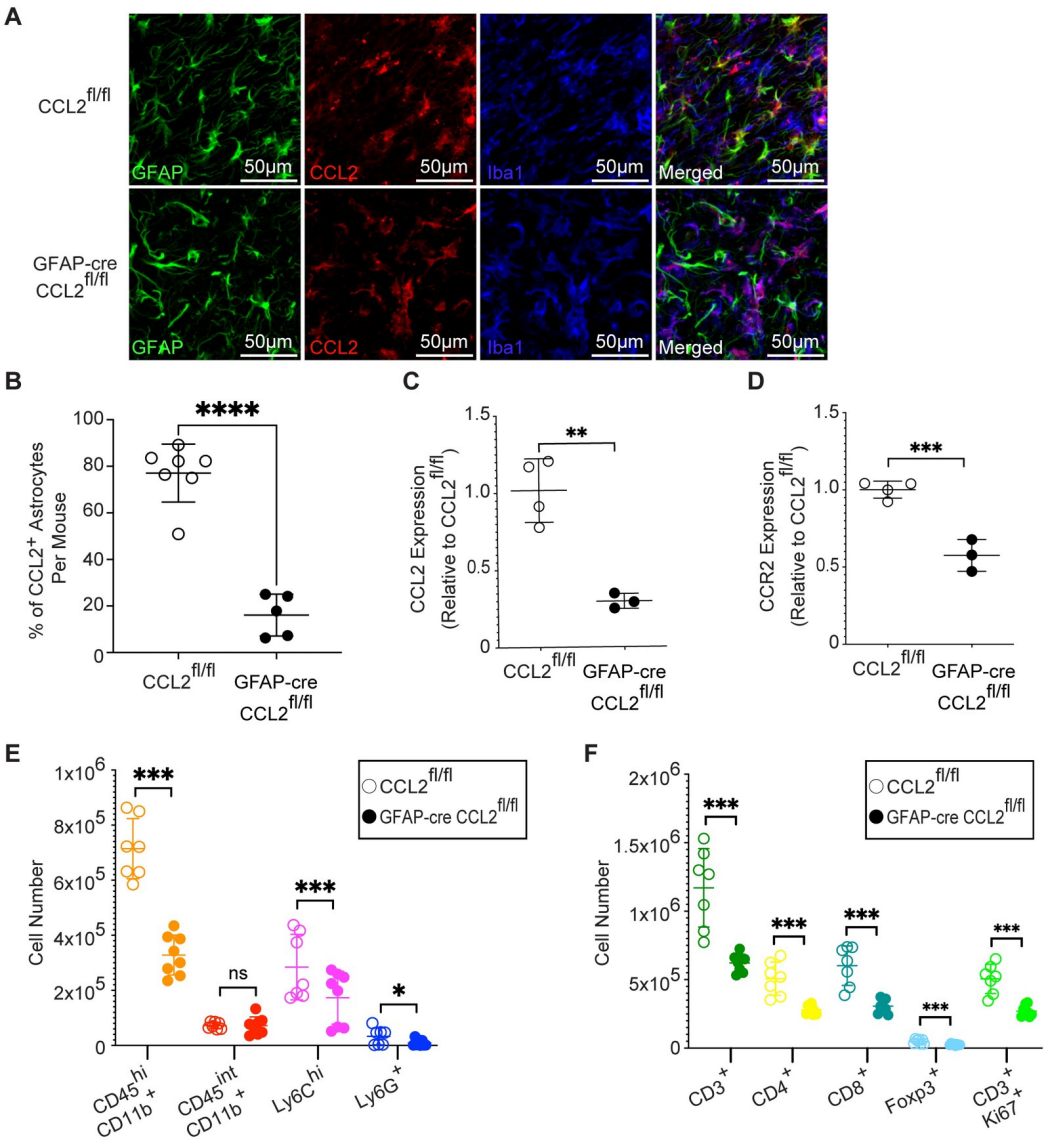
Astrocyte-derived CCL2 drives immune cell recruitment to the brain during chronic *T*. *gondii* infection. CCL2^fl/fl^ and GFAP-cre CCL2^fl/fl^ mice were infected with *T*. *gondii* and examined at 28 DPI. (A) Representative confocal microscopy of GFAP^+^ astrocytes (green), CCL2-RFP (red), and Iba1^+^ myeloid cells (blue) in CCL2^fl/fl^ and GFAP-cre CCL2^fl/fl^ mice at 28 DPI with PRU strain. (B) Percent of CCL2^+^ GFAP^+^ cells of total GFAP^+^ cells per FOV was averaged for each mouse. (C and D) qPCR for *ccl2* (C) or *ccr2* (D) transcripts from brains of CCL2^fl/fl^ and GFAP-cre CCL2^fl/fl^ mice at 28 DPI with ME49 strain. Transcripts are normalized to *gapdh* and shown relative to the mean transcript level of the CCL2^fl/fl^ mice. (E) Quantification of brain myeloid immune cells by flow cytometry of CCL2^fl/fl^ and GFAP-cre CCL2^fl/fl^ mice at 28 DPI with ME49 strain. (F) Quantification of brain T cells by flow cytometry at 28 DPI with ME49 strain. In (B) *n* = 5–7 mice per group from three experiments. In (C and D) *n* = 3–4 mice per group, and in (E and F) *n* = 7–8 mice per group from two experiments. Statistical significance was determined by Student’s *t*-test (B-D) or randomized block ANOVA (E-F). *p<0.05, **p<0.01, ***p<0.001, ****p<0.0001, ns: not significant.

We next aimed to determine the importance of astrocyte-derived CCL2 during chronic infection when parasites have converted into the slow growing bradyzoites within cysts, and astrocytes comprise the majority of CCL2 producing cells [38]. We infected GFAP-Cre CCL2^fl/fl^ and control CCL2^fl/fl^ mice as above and euthanized the mice at 28 DPI. CCL2-RFP was detected in control mice during chronic infection and was significantly reduced in astrocytes in GFAP-Cre CCL2^fl/fl^ mice (Fig 5A and 5B). To control for the specificity of CCL2 deletion, we confirmed that there was no reduction in CCL2-RFP signal in Iba-1^+^Mac2^-^ microglia, Mac2^+^ myeloid cells, or NeuN^+^ neurons in the GFAP-Cre CCL2^fl/fl^ mice (S7A–S7C Fig), as expected. Since astrocytes are the major producers of CCL2 during chronic infection, we examined the extent to which astrocytes contribute to overall CCL2 production in the brain at this timepoint. We measured *ccl2* expression in the brain using real-time qPCR and detected a 70% decrease in *ccl2* mRNA levels in the brains of the knockout mice during infection (Fig 5C).

To determine how deficiency in astrocyte-derived CCL2 affects recruitment of CCR2^+^ cells to the brain, *ccr2* transcripts in the brain were examined at 28 DPI. Unlike during acute infection, the deficiency in astrocyte-derived CCL2 led to a 50% decrease in *ccr2* mRNA levels in the brain (Fig 5D). To identify the immune cell subsets affected by this decrease in CCL2 levels during chronic *T*. *gondii* infection, we examined myeloid cells, granulocytes, and T cells in the brain. There was no change in the frequencies of microglia (CD45^int^ CD11b^+^) in the absence of astrocyte-derived CCL2, as expected. However, the mobilization of infiltrating myeloid cells (CD45^hi^ CD11b^+^), inflammatory monocytes (CD45^hi^ Ly6C^hi^), and neutrophils (Ly6G^+^) all declined in the brains of the knockout mice (Figs 5E and S8). We also detected reductions in T cell numbers in the brain, including helper T cells (CD4^+^), cytotoxic T cells (CD8^+^), regulatory T cells (Foxp3^+^), and proliferating T cells (Ki67^+^) (Fig 5F), indicating a role for astrocyte-derived CCL2 in mobilizing these cells to the brain during chronic infection. In contrast, in the meninges there was a difference in the number of infiltrating monocytes, but not of granulocytes or T cells in the knockout compared to the control mice (S5F Fig). Additionally, the frequencies of immune cells in the spleen were unaffected by deficiency in astrocyte-derived CCL2, as expected (S5E Fig).

**Fig 6:**
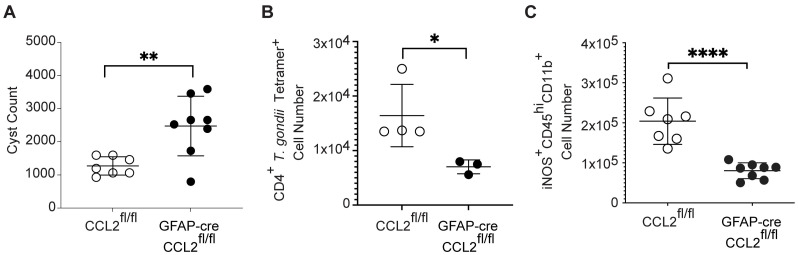
Mice deficient in astrocyte-derived CCL2 have reduced parasite control and decreased immune defense during chronic *T*. *gondii* infection. CCL2^fl/fl^ and GFAP-cre CCL2^fl/fl^ mice were infected with ME49 strain *T*. *gondii* and examined at 28 DPI. (A) *T*. *gondii* cyst counts in brains from infected mice at 28 DPI. (B) Quantification of *T*. *gondii* tetramer^+^ CD4^+^ T cells in the brains of infected mice by flow cytometry at 28 DPI. (C) Quantification of iNOS^+^ myeloid cells by flow cytometry of the brains of infected mice at 28 DPI. In (A and C) *n* = 7–8 mice per group from 2 experiments, and in (B) *n* = 3–4 mice per group from 1 experiment. Statistical significance was determined by a randomized block ANOVA. *p<0.05, **p<0.01, ***p<0.001.

Consistent with prior studies demonstrating that CCR2^+^ cells are important for the control of *T*. *gondii* infection in the brain [27], the reduction in CCR2^+^ immune cell infiltration was associated with a doubling of the total number of cysts in the brain (Fig 6A). However, the knockout mice did not exhibit impaired survival or increased weight loss out to 28 DPI (S9A and S9B Fig). These results indicate that astrocyte-derived CCL2 plays a critical role in the control of *T*. *gondii* burden, but not necessarily survival, during chronic infection of the brain.

To determine potential mechanisms explaining the reduced control of *T*. *gondii* infection, we analyzed key antimicrobial and immune mechanisms of host defense. As T cells are required for controlling *T*. *gondii* infection, we measured *T*. *gondii* AS15 tetramer-specific T cells [47]. We detected decreased levels of CD4^+^ *T*. *gondii* tetramer^+^ cells in the brain (Fig 6B). iNOS is increased in myeloid cells during *T*. *gondii* infection and plays a critical role in the control of the parasites during chronic infection [23][26]. To determine if this antimicrobial pathway was affected in mice deficient in astrocyte-derived CCL2, we measured iNOS^+^ myeloid cells using flow cytometry and detected a decrease in the number of these cells in the knockout compared to the control mice during infection (Fig 6C). These data demonstrate that during chronic infection, astrocyte-derived CCL2 plays a key role in driving parasite-controlling immune cells to the brain.

## Discussion

Neuroinflammatory responses must be tightly regulated to enable sufficient immune cell infiltration to the brain during infection but limit excessive immunopathology, which is correlated with neuroinflammatory and neurodegenerative diseases. As toxoplasmic encephalitis continues to account for AIDS-related deaths [14], the drivers of immune cell mobilization to the brain during *T*. *gondii* infection warrant further investigation. Prior studies have shown that CCL2 is necessary for host immune protection against *T*. *gondii* infection [28]. However, whole-body CCL2 knockout mice have reduced levels of circulating immune cells, since CCL2 is required for monocyte egress from the bone marrow. In the brain, CCL2 is correlated with immune cell recruitment, but the role of specific CCL2-producing cells in the brain during *T*. *gondii* infection is unknown [38]. Our study found that astrocyte-derived CCL2 plays a pivotal role during chronic, but not acute, *T*. *gondii* infection in the brain.

In examining the upregulation of CCL2 in the brain during acute *T*. *gondii* infection, we found that CCL2-expressing cells were not uniformly dispersed throughout the brain. Rather, clusters of CCL2-producing cells were detectable in the cerebrum, frequently surrounding *T*. *gondii* tachyzoites. As monocyte infiltration and localization in the brain is highly associated with parasite clusters [31], *T*. *gondii* infection may be a direct driver of CCL2 production, thereby inducing this region-specific monocyte recruitment in the brain. *T*. *gondii* is known to trigger CCL2 production in infected host cells through the activities of the dense granule protein GRA25 [48]. However, it is also clear that direct invasion of host cells in the brain by *T*. *gondii* is not required for CCL2 production. Merritt *et al*. found that CCL2 expression is upregulated in regions of the brain cells without significant interaction with *T*. *gondii*, but was found within 120 μm of a parasite-interacted cell [49]. In the periphery, innate immune recognition of *T*. *gondii* profilin by TLR11/12 induces CCL2 production and monocyte recruitment [33]. The production of CCL2 in the periphery and in human monocytes is also regulated by S100A11 in a RAGE-dependent manner [34]. Therefore, *T*. *gondii* likely induces a localized upregulation of CCL2 in the brain through a combination of parasite and host factors. Additionally, the nonuniform distribution of CCL2 across the brain may explain why astrocyte-derived CCL2 was more important for recruiting immune cells to the brain parenchyma than to the meninges. These data suggest that CCL2 production by specific cells in the brain may vary in importance depending on its location.

Interestingly, when we analyzed CCL2 production from acute to chronic infection, we found that the major CCL2-producing cell type transitioned from myeloid cells to astrocytes. It is possible that the cues driving CCL2 production during acute infection differ from those during chronic infection, particularly since the composition of immune cells and cytokines in the brain changes as the parasites undergo stage conversion from rapidly replicating tachyzoites to slow-growing bradyzoites that are encysted within neurons. Microglia activation by TNF-α induces CCL2 production and monocyte recruitment to the brain in a model of hepatic inflammation [50]. By 10 days after *T*. *gondii* infection, ILCs are recruited to the brain, and these cells are correlated with TNF-α and CCL2 expression in the brain [51]. Therefore, during acute *T*. *gondii* infection, TNF-α production by ILCs may be inducing CCL2 production in microglia to recruit the monocytes to the brain. In contrast, during chronic infection, CCL2 production by astrocytes is induced by the IL-33 alarmin in close proximity to *T*. *gondii* [38,52]. Notably, the ability of astrocytes, but not myeloid cells, to recognize IL-33 via its receptor ST2 is key for CCL2 production, immune cell recruitment to the brain, and parasite control during chronic, but not acute infection [38]. Therefore, the changes in the neuroimmune landscape between acute and chronic infection likely contribute to distinct cues and cell types producing CCL2 at different stages of the infection.

In infected astrocyte-specific CCL2 knockout mice, *ccl2* expression was more significantly reduced during chronic infection compared to acute infection, and a decline in monocytes, neutrophils, and multiple subsets of T cells in the brain was only observed during chronic infection. These data suggest that the reduction in *ccl2* during acute infection of the knockout mice was not sufficient to impact immune infiltration of the brain, or alternatively, that during acute infection, other chemokines orchestrate the initial recruitment of immune cells to the brain. Investigating a role for microglia-derived CCL2 in early immune cell recruitment to the brain would necessitate the use of a microglia-specific Cre driver mouse line. There are challenges with this strategy, since many markers for microglia are also expressed by peripheral myeloid cells, including monocytes and macrophages. However, an inducible system, such as the CX3CR1-CreERT2 model has proven successful for inducing reporter gene expression specifically in microglia during *T*. *gondii* infection [43]. Crossing these mice to mice with floxed *ccl2* may be a promising approach for analyzing the importance of CCL2 production by microglia in the control of *T*. *gondii* infection in the brain.

One surprising observation was the effect of astrocyte-specific CCL2 deficiency on immune cells that do not express the CCR2 receptor during *T*. *gondii* infection, including T cells and neutrophils. It is likely that this effect is indirect: during *T*. *gondii* infection, astrocyte-derived CCL2 recruits CCR2^+^ monocytes, which along with brain-resident cells, may produce chemokines that recruit non-CCR2-expressing T cells and neutrophils, as proposed in other models [53]. However, unlike in the brain, Ly6C^+^ monocyte infiltration of the meninges was reduced in mice deficient in astrocyte-derived CCL2 during chronic infection. These findings are notable, since astrocytes comprise the glia limitans, a thin barrier that separates the brain parenchyma from the pia mater of the meninges. These data suggest that astrocytes in the glia limitans contribute to CCL2 production, such that knocking out CCL2 in astrocytes impacts meningeal monocyte infiltration. These findings also indicate that other chemokines produced within the meninges may be the primary chemoattractants for T cell and granulocyte infiltration of the meninges.

Although the decreased immune cell mobilization to the brains of GFAP-cre CCL2^fl/fl^ mice was accompanied with a near doubling of cysts in the brain compared to control CCL2^fl/fl^ mice, the survival and weight loss of the mice did not differ between the two genotypes. While a doubling of parasite cysts represents a substantial increase in parasite burden, it may not be sufficient to affect survival of the mice. It is clear that CCR2 blockade in the chronic phase of infection is fatal [27]. Astrocyte CCL2 reduces the infiltration of CCR2^+^ monocytes, but not to a degree that is fatal, suggesting that other sources of CCL2 maintain sufficient numbers of protective immune cells to provide a degree of host resistance against the parasite.

Collectively, these data describe the significance of astrocyte-derived CCL2 on the neuroimmune environment over the course of *T*. *gondii* infection. This study finds that the main producers of CCL2 vary based on the stage of infection in the brain–myeloid cells are key during acute infection and astrocytes during chronic infection–and defines a role for CCL2 from astrocytes in inducing immune infiltration and decreasing parasite burden during chronic, but not acute, infection. An understanding of cell type-specific CCL2 production may ultimately enable the targeting of this chemokine for enhancing or inhibiting immune cell recruitment to the brain during specific stages of neuroinflammatory and neurodegenerative diseases.

## Materials and methods

### Ethics statement

All procedures and protocols were approved by the University of California, Irvine’s Institutional Animal Care and Use Committee protocol number AUP-21-105 and the University of Virginia’s Institutional Animal Care and Use Committee protocol number 3968.

### Experimental mice

C57BL/6 (Jackson stock No: 000664), CCR2^RFP/RFP^ (Jackson stock No: 017586) CCL2-RFP^fl/fl^ (Jackson Stock No: 016849), and GFAP-Cre (Jackson Stock No. 024098) mice were purchased from Jackson laboratories. We bred GFAP-Cre homozygous mice to CCL2-RFP^fl/fl^ mice, in which the *ccl2* gene is fused to cDNAs for *HA* and *mCherry (RFP)*, separated by a self-cleaving aphthovirus 2A cleavage site and flanked by *loxP* sites [40]. Therefore, CCL2-producing cells, rather than CCL2 itself, are labeled with RFP. These mice are referred to as CCL2-RFP and CCL2^fl/fl^ mice in this manuscript. The GFAP-Cre and CCL2^fl/fl^ mice were confirmed using genotyping on ear punches. To confirm that these mice were not germline knockouts, we conducted microscopy, qPCR, or ELISA for CCL2 on brain tissue and detected CCL2 in non-astrocytes in all mice. CCR2^RFP/+^ were generated by breeding CCR2^RFP/RFP^ mice with C57BL/6J wildtype mice. Mice were infected with *T*. *gondii* at 6 to 14 weeks of age and were housed separately from breeding animals. At necropsy, mice were anesthetized via intraperitoneal injection of 2.5% Tribromoethanol (Avertin, Sigma Aldrich) and transcardially perfused with 50 mL of 1X PBS (Corning) to remove non-adherent blood cells.

### Parasite strains

Mice were infected intraperitoneally with 200 type II *Toxoplasma gondii Prugniaud* strain tachyzoites or 10 ME49 cysts in 200 μL of 1X PBS. Uninfected control mice were injected intraperitoneally with 200 μL of 1X PBS. Tachyzoites were maintained via serial passage in human foreskin fibroblasts, as described previously [54]. *T*. *gondii* tissue cysts were obtained from infected Swiss Webster mice.

### Flow cytometry

To isolate single cells from mouse brains, the harvested brains were minced and digested using dispase II (Toche Applied Science) diluted in Hepes-buffered saline. To filter out clumps, the mixture was triturated then passed through a 70 μm filter (Falcon), and myelin was removed using a 35% and 75% percoll (GE Healthcare) gradient. Alternatively, cells were isolated by mincing brains in RPMI with 10% FBS (R10). The brains were then homogenized using a 3 mL syringe and 18-gauge needle and digested in a 2 mL mixture of collagenase-dispase (Sigma), and DNAse I (ThermoFisher) in R10. The mixture was triturated then passed through a 70 μm filter (Falcon), and myelin was removed using a spin in 40% percoll. To isolate single cells from spleens, spleens were homogenized and passed through a 40 μm filter (Falcon), and red blood cells were lysed used ACK lysing buffer (Gibco). To isolate single cells from meninges, the dura was collected from the skull and digested using collagenase D (Roche) and DNase I (Thermo Scientific), and the tissue was passed through a 70 μm filter (Falcon).

Single cell suspensions were resuspended in 10% TrueStain FcX (Biolegend) in staining buffer (3% fetal bovine serum in 1X PBS) to inhibit nonspecific antibody binding to the cells. The cells were then surface-stained with fluorescent dye-conjugated antibodies diluted in staining buffer for 30 minutes, washed, then fixed in 2% paraformaldehyde for 5 minutes. The following reagents from eBioscience were used: fixable viability dye eFluor 506 Cat#65-0866-18, CD3:FITC Cat#11-0031-85, CD8α:PerCp-Cy5.5 Cat#45-0081-82, CD4:PE-Cyanine-7 Cat#25-0041-82, CD45:PerCp-Cy5.5 Cat#45-0451-80, Ly6C:PE-Cyanine-7 Cat#25-5932-82, CD11b:AF780 Cat#47-0012-82; from BD biosciences: CD45.2 FITC Cat#561874; from Biolegend: Ly6G:BV510 Cat#127633, CD11b:BV605 Cat#101257, CD45:BV785 Cat#103149, Ly6C:PerCp-Cy5.5 Cat#128017, CD3:APC-Cy7 Cat#100222, CD206:PE Cat#141706, F4/80:APC Cat#123116. For intracellular staining, cells were fixed for 30 min at 4°C with a fixation/permeabilization kit (eBioscience Cat#00-5123-43 and Cat#00-5223-56). Samples were then stained intracellularly with antibodies in permeabilization buffer (eBioscience Cat#00-8333-56) for 30 min at 4°C (eBioscience: Ki67 APC Cat#17569880, iNOS APC Cat#17-5920-80, Foxp3 PB Cat#48-5773-82). For intracellular cytokine staining, cells were prepared as above but treated with Brefeldin A (Biolegend) diluted in D10 media for 4 hours prior to incubation in 10% TrueStain FcX and extracellular surface staining. Cells were then fixed and permeabilized with the BD Cytofix/Cytoperm solution kit (BD Biosciences Scientific), then stained with primary rabbit anti-RFP antibody (Rockland Cat#600-401-379) followed by goat anti-rabbit:AF647 secondary antibody (Invitrogen). Following staining, samples were resuspended in 1X PBS (Corning) and run on the Novocyte flow cytometer (Agilent). Subsequently, the data were analyzed utilizing FlowJo software (Treestar).

### Immunohistochemistry

After perfusion, brains were removed and placed in 4% paraformaldehyde for 4–12 hours at 4°C and in 30% sucrose in 1X PBS at 4°C until the brain tissues sank. The brains were embedded in O.C.T. compound (Fisher), frozen on dry ice, and stored at -80°C. 14 μm-thick sections were cut and directly mounted onto superfrost plus microscopy slides (Fisherbrand) using a HM525 NX Cryostat (Thermo Scientific). Sections were dried at 37°C then placed in 1X PBS to remove the O.C.T. The sections were incubated at room temperature with primary antibodies (goat anti-mouse Iba-1 Abcam Cat#5076 1:50, rat anti-mouse GFAP Invitrogen Cat#130300 1:300, rabbit anti-RFP Rockland Cat#600-401-379 1:500, biotinylated mouse anti-mouse NeuN Sigma Cat# MAB377B 1:200, or biotin rat anti-mouse Mac2 Cedarlane Labs Cat# CL8942AP 1:100), 3% donkey serum, and 0.3% Triton-X100 diluted in 1X PBS overnight. Sections were washed the next day with 1X PBS and incubated at room temperature with secondary antibodies (donkey anti-goat Abcam Cat# ab175665 1:500, donkey anti-rat Jackson Immunoresearch Cat#712-606-153 1:500, donkey anti-rabbit Jackson Immunoresearch Cat#711-585-152 1:500, or streptavidin conjugated to AF647 Thermofisher Scientific S32357 1:500), 3% donkey serum, and 0.3% Triton-x100 diluted in 1X PBS overnight. Sections were then washed with 1X PBS and autoclaved water, and mounted using antifade mounting media (Vectashield) and covered with microscope cover glass (Fisherbrand). Slides were stored at 4°C. Images were taken in the cerebrum, interbrain, and midbrain with a TCS SP8 confocal microscope (Leica) and analyzed using ImageJ software.

### qPCR

*Genomic DNA*: Brains were minced, and DNA was extracted using the DNA/RNA Mini Kit (Qiagen). Brain tissue was used in qPCR with primers targeting the *T*. *gondii*-specific B1 gene (forward primer 5’-CAGATGTGCTAAAGGCGTCA-3’, reverse primer 5’-GCCCTAGACAGACAGCGAAC-3’) and results were normalized to the mouse glyceraldehyde-3-phosphatase-dehydrogenase (GAPDH) gene (forward primer 5’-GCATGGCCTTCCGTGTTC-3’, reverse primer 5’-CCCAGCTCTCCCCATACATA-3’).

*mRNA*: Brain tissue was minced, and RNA was extracted using the DNA/RNA Mini Kit (Qiagen), or by placing 100 mg of brain tissue into bead-beating tubes (Sarstedt, Cat# 72.693.005) with 1 mL Trizol (Ambion, Cat#15596018) and zirconia/silica beads (Biospec, Cat#11079110z). The brain was homogenized for 30 seconds with a Mini-bead beater (Biospec) machine, and Trizol was used to extract RNA. cDNA was generated using a High-Capacity Reverse Transcription Kit (Applied Biosystems, Cat#4368813). cDNA was also generated by treating RNA with DNase I (Invitrogen 18068015) and incubation of the RNA with random hexamers (Invitrogen Cat#N8080127) and 10 mM dNTPs (Invitrogen Cat#18427013). The final product was divided into two tubes. One tube was treated with 10X RT buffer, 25 mM MgCl_2_ (ThermoFisher Scientific Cat#R0971), 0.1 M DTT (Thermofisher Scientific Cat#P2325), 40 U/μL RNase OUT (Invitrogen Cat#10777019), and 200 U/μL Superscript III RT (Invitrogen Cat#18080093) to generate cDNA. The other tube was treated similarly but without reverse transcriptase as a control. RNase H (Thermofisher Scientific Cat#EN0202) was added to remove RNA in RNA/DNA hybrids. Transcripts were measured using RT-qPCR targeting the *ccl2* gene (forward primer 5’- TCTCTTCCTCCACCACCATG -3’, reverse primer 5’-CTCCAGCCTACTCATTGGGA-3’) or *ccr2* gene (forward primer 5’-CAAATCAAAGGAAATGGAAGACAAT-3’, reverse primer 5’- GCCCCTTCATCAAGCTCTTG -3’), and results were normalized to the mouse *gapdh* gene (forward primer 5’- GCATGGCCTTCCGTGTTC -3’, reverse primer 5’- GATGTCATCATACTTGGCAGGTTT -3’).

qPCR was run on the CFX Opus 384 Real-Time PCR System using the iTaq Universal SYBR green supermix (Bio-Rad). The outputted values were analyzed using the cycle threshold (-2^(ΔΔCT)^) method [55]. Gene expression was also measured using *ccl2* (Applied Biosystems Cat#Mm00441242_m) or *ccr2* (ThermoFisher Mm04207877_m1) gene expression assays (and a 2X Taq-based mastermix (Bioline, Cat#BIO-86005) on a CFX384 Real-Time System (Bio-Rad). Samples were normalized to *hprt* (Applied Biosystems, Cat#Mm00446968_m1), and relative expression to controls was calculated as 2^(-ΔΔCT)^.

### ELISA

Brains were snap frozen, homogenized using a mortar and pestle, and resuspended in 1X PBS with protease and phosphatase inhibitor (Thermo Scientific Cat#78444). Cells were centrifuged to pellet debris, and the supernatant was used for ELISA with the Mouse MCP-1 (CCL2) ELISA max kits from Biolegend (Cat#432704). Samples were run in triplicate and analyzed on a Spectra Max Plus 384 nm (Molecular Devices) spectrophotometer.

### *T*. *gondii* cyst counts

100 mg of brain tissue was minced in 2 mL complete RPMI. The tissue was then passed through a 18-gauge and a 22-gauge needle. 30 μL of the final brain homogenate was mounted on a microscope slide, and cysts were enumerated using a Brightfield DM 2000 LED microscope (Leica). Total cyst burden of the full brain was deduced from these numbers.

### Statistical analyses

Statistics were conducted using *t*-test, ANOVA, or randomized block ANOVA (on two groups at identical time points). Graphs were generated in Prism using software version 9.3.1. The number of samples per group and statistical test utilized can be found in the figure legend for each figure. Significance was represented as follows: ns = not significant, *p<0.05, **p<0.01, ***p<0.001, ****p<0.0001. Error bars in all figures represent standard deviations.

## Supporting information

S1 Fig Ly6C^+^ and Ly6B.2^+^ immune cells but not NeuN^+^ neurons produce CCL2 during *T*. *gondii* infection. CCR2-RFP mice were injected with PBS or infected with *T*. *gondii* (PRU strain), and at 15 DPI brain sections were stained with antibodies and imaged using confocal microscopy. (A) Representative images of *T*. *gondii* (white), Ly6C^+^ cells (green), CCL2-RFP (red), and CD31^+^ cells (blue). Note that the anti-Ly6C antibody stains some CD31^+^ blood vessels in addition to staining infiltrating monocytes. (B) Representative images of *T*. *gondii* (white), Ly6B.2 ^+^ infiltrating cells (green), CCL2-RFP (red), and Iba1^+^ myeloid cells (blue). (C) Representative images of *T*. *gondii* (white), NeuN ^+^ neurons (green), CCL2-RFP (red), and DAPI^+^ nuclei (blue) in FOV with or without parasites at 15 DPI.
(TIF)

S2 Fig Gating strategies for immune cells in the brain and meninges. Gates were drawn based on the fluorescence minus one (FMO) controls for the brains and meninges. (A) Representative flow cytometry gating scheme of brain cells from PBS-injected (top) or PRU strain *T*. *gondii-*infected (bottom) CCL2-RFP mice at 15 DPI. (B) Representative flow cytometry gating scheme of meningeal cells isolated from PBS-injected (top) or *T*. *gondii-*infected (bottom) CCL2-RFP mice at 15 DPI.
(TIF)

S3 Fig CCL2-RFP expression in myeloid cells in the brain. CCL2-RFP mice were injected with PBS as a control or infected with PRU strain *T*. *gondii*, and brains were harvested at 15 DPI. Immune cells from the brain homogenates were analyzed by flow cytometry, and the mean fluorescence intensity (MFI) of CCL2-RFP in CCL2-RFP^+^ cells from PBS-injected (open circles) or *T*. *gondii*-infected (closed circles) mice was determined. *n* = 4–9 mice per group from three experiments. Statistical significance was determined by randomized block ANOVA. **p<0.01, ns: not significant.
(TIFF)

S4 Fig Genotyping of mice. (A) Gel from PCR of genomic DNA isolated from ear punches showing the presence of floxed *ccl2* (top band labeled “Mutant CCL2”) in GFAP-Cre CCL2^fl/fl^, CCL2^fl/fl^, and GFAP-Cre CCL2^fl/+^ mice but not in C57BL/6 mouse. Endogenous *ccl2* locus without loxP sequences (bottom band) is detected in C57BL/6 mice and GFAP-Cre CCL2^fl/+^mice, but not in GFAP-Cre CCL2^fl/fl^ nor CCL2^fl/fl^ mice. (B) Gel from PCR showing the presence of *cre* (top band) in GFAP-Cre CCL2^fl/fl^ and GFAP-Cre CCL2^fl/+^ mice but not in CCL2^fl/fl^ nor C57BL/6J wildtype mice. To control for the presence of DNA in each sample, primers to detect the T cell receptor (TCR) (bottom band) were used.
(TIF)

S5 Fig Immune cell frequencies in the spleen and meninges of GFAP-Cre CCL2^fl/fl^ mice during infection. Control CCL2^fl/fl^ (open circles) and GFAP-Cre CCL2^fl/fl^ (closed circles) mice were injected with PBS (A and B) or infected with *T*. *gondii* (PRU 15 DPI, and ME49 28 DPI) (C-F), and spleens and meninges were harvested. (A, C, E) Frequencies of spleen CD11b^+^ myeloid cells, Ly6C^hi^ monocytes, Ly6C^lo^ monocytes, Ly6G^+^ neutrophils, CD3^+^ T cells, CD4^+^ T cells, and CD8^+^ T cells by flow cytometry. (B, D, F) Frequencies of meningeal F4/80^+^CD206^+^ macrophages, Ly6C^+^ monocytes, Ly6G^+^ neutrophils, and CD3^+^ T cells. In (A and B) *n* = 4–5 mice per group, in (C and D) *n* = 4–7 mice per group, in (E) *n* = 3–4 mice per group, and in (F) *n* = 6–8 mice per group from at least two independent experiments. Statistical significance was determined by a randomized block ANOVA. *p<0.05, ns: not significant.
(TIF)

S6 Fig Identity of CCR2-RFP^+^ cells in the brain during *T*. *gondii* infection. CCR2^RFP/+^ mice were injected i.p. with 200 *T*. *gondii* (PRU strain), and brains were harvested at 15 DPI for flow cytometry of single cells. The percent of CCR2-RFP^+^ cells from each immune cell population is plotted. *n* = 7 mice per group. Statistical significance was determined by a one-way ANOVA. ****p<0.0001.
(TIF)

S7 Fig CCL2 production by NeuN^+^ neurons and Iba1^+^and Mac2^+^ myeloid cells in GFAP-Cre CCL2^fl/fl^ and CCL2^fl/fl^ mice. CCL2^fl/fl^ and GFAP-cre CCL2^fl/fl^ mice were infected with *T*. *gondii* (PRU strain) and the brains were harvested and stained with antibodies for analysis at 28 DPI. (A) Representative confocal microscopy of NeuN^+^ neurons (green), CCL2-RFP (red), and DAPI (blue). (B) Representative confocal microscopy of Mac2^+^ myeloid cells, (green), CCL2-RFP (red), and Iba1^+^ myeloid cells (blue). (C) Percent area of CCL2-RFP signal within each cell type. *n* = 12–23 FOV from 5–7 mice per group from 2 experiments. Statistical significance was calculated using Student’s *t*-test. ns, not significant.
(TIF)

S8 Fig Frequencies of myeloid cells in the brains of GFAP-Cre CCL2^fl/fl^ and CCL2^fl/fl^ mice during chronic *T*. *gondii* infection. CCL2^fl/fl^ or GFAP-cre CCL2^fl/fl^ mice were infected with *T*. *gondii* (ME49 strain), and the brains were harvested at 28 DPI. The frequencies of myeloid immune cells in the brain were determined by flow cytometry. *n* = 7–8 mice per group from two experiments. Statistical significance was determined by randomized block ANOVA. *p<0.05, **p<0.01, ns, not significant.
(TIF)

S9 Fig Survival and weight loss of GFAP-Cre CCL2^fl/fl^ and CCL2^fl/fl^ mice during *T*. *gondii* infection. CCL2^fl/fl^ and GFAP-cre CCL2^fl/fl^ mice were infected with *T*. *gondii* (PRU strain) and monitored for 28 DPI. **(A)** Gehan-Breslow-Wilcoxon survival curves were generated for CCL2^fl/fl^ (red) and GFAP-Cre CCL2^fl/fl^ (black) mice. *n* = 10 mice per group from two experiments. **(B)** Weight loss curves were generated for CCL2^fl/fl^ (red) and GFAP-Cre CCL2^fl/fl^ (black) mice. *n* = 10 mice per group from three experiments. Statistical significance between the slopes of the curves were measured between 0 and 7 DPI, 7 and 14 DPI, 14 and 21 DPI, and 21 to 28 DPI using linear regression. ns, not significant.
(TIF)
