# Supplementary figures and images for: Deficiency in astrocyte CCL2 production reduces neuroimmune control of Toxoplasma gondii infection
Source: PLoS Pathog. 2024 Jan 11;20(1):e1011710. doi: 10.1371/journal.ppat.1011710 (PMC10807779; doi:10.1371/journal.ppat.1011710)

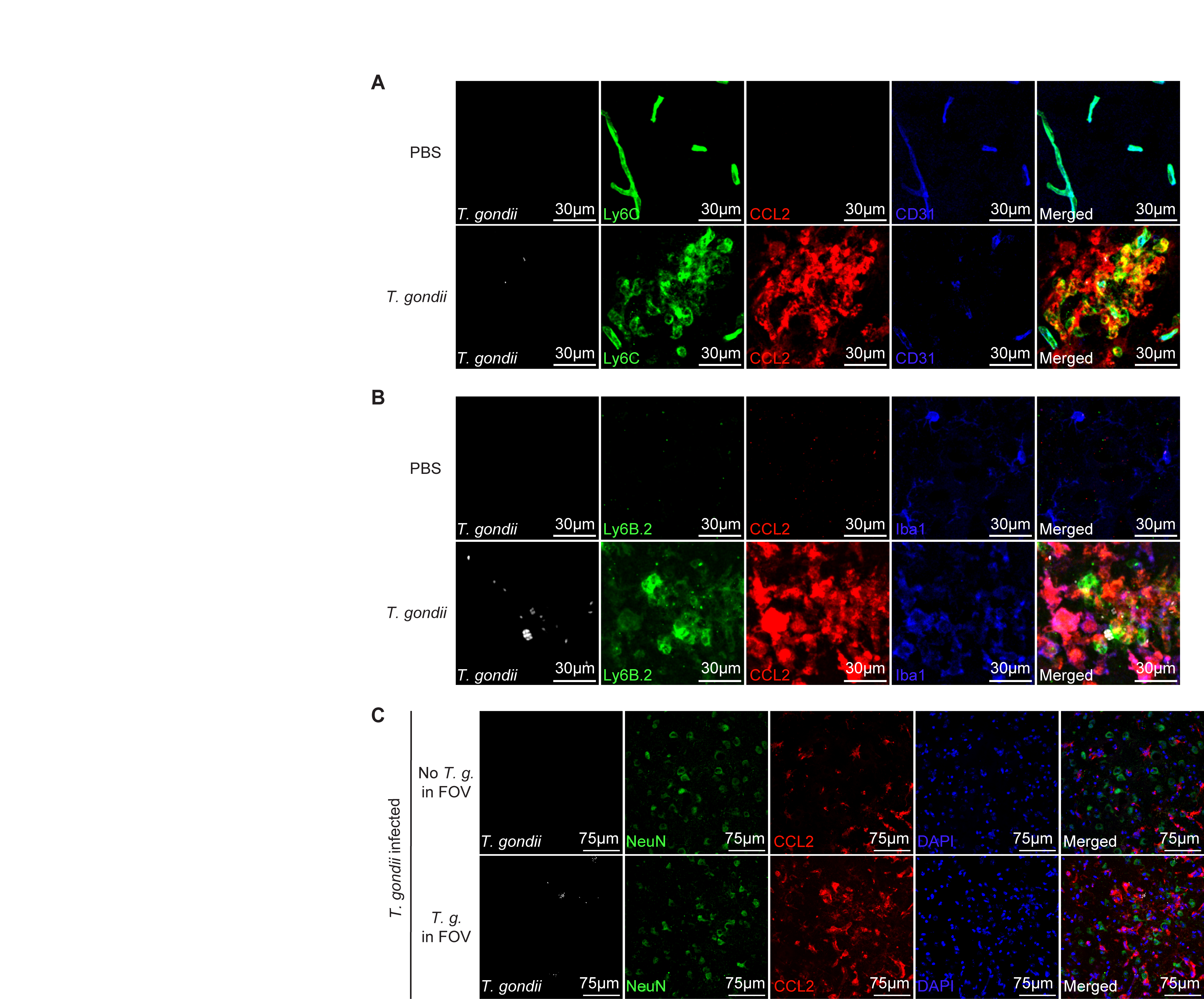

Supplement: S1 Fig — CCR2-RFP mice were injected with PBS or infected with T. gondii (PRU strain), and at 15 DPI brain sections were stained with antibodies and imaged using confocal microscopy. (A) Representative images of T. gondii (white), Ly6C+ cells (green), CCL2-RFP (red), and CD31+ cells (blue). Note that the anti-Ly6C antibody stains some CD31+ blood vessels in addition to staining infiltrating monocytes. (B) Representative images of T. gondii (white), Ly6B.2 + infiltrating cells (green), CCL2-RFP (red), and Iba1+ myeloid cells (blue). (C) Representative images of T. gondii (white), NeuN + neurons (green), CCL2-RFP (red), and DAPI+ nuclei (blue) in FOV with or without parasites at 15 DPI. (TIF) [file ppat.1011710.s001.tif]

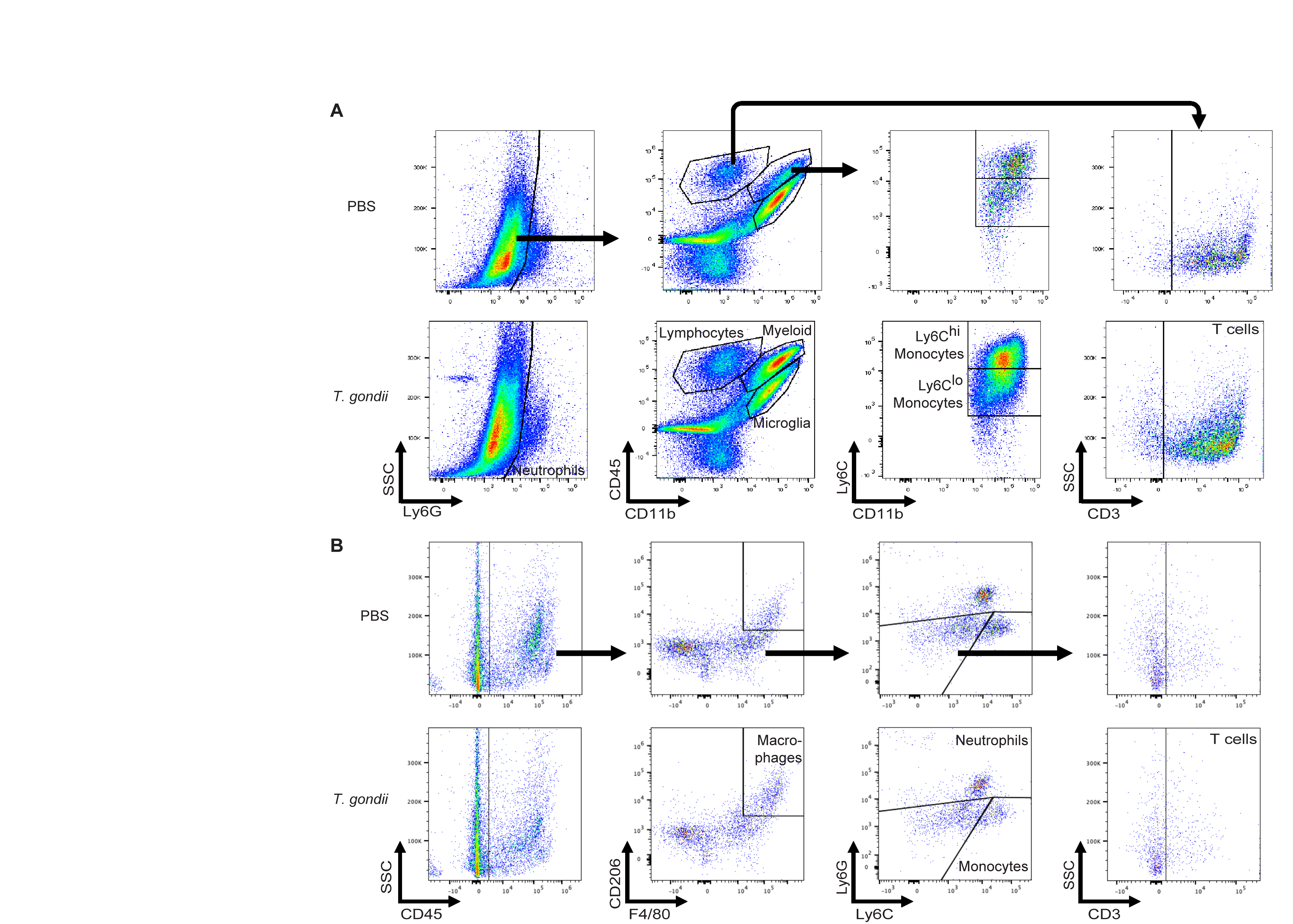

Supplement: S2 Fig — Gates were drawn based on the fluorescence minus one (FMO) controls for the brains and meninges. (A) Representative flow cytometry gating scheme of brain cells from PBS-injected (top) or PRU strain T. gondii-infected (bottom) CCL2-RFP mice at 15 DPI. (B) Representative flow cytometry gating scheme of meningeal cells isolated from PBS-injected (top) or T. gondii-infected (bottom) CCL2-RFP mice at 15 DPI. (TIF) [file ppat.1011710.s002.tif]

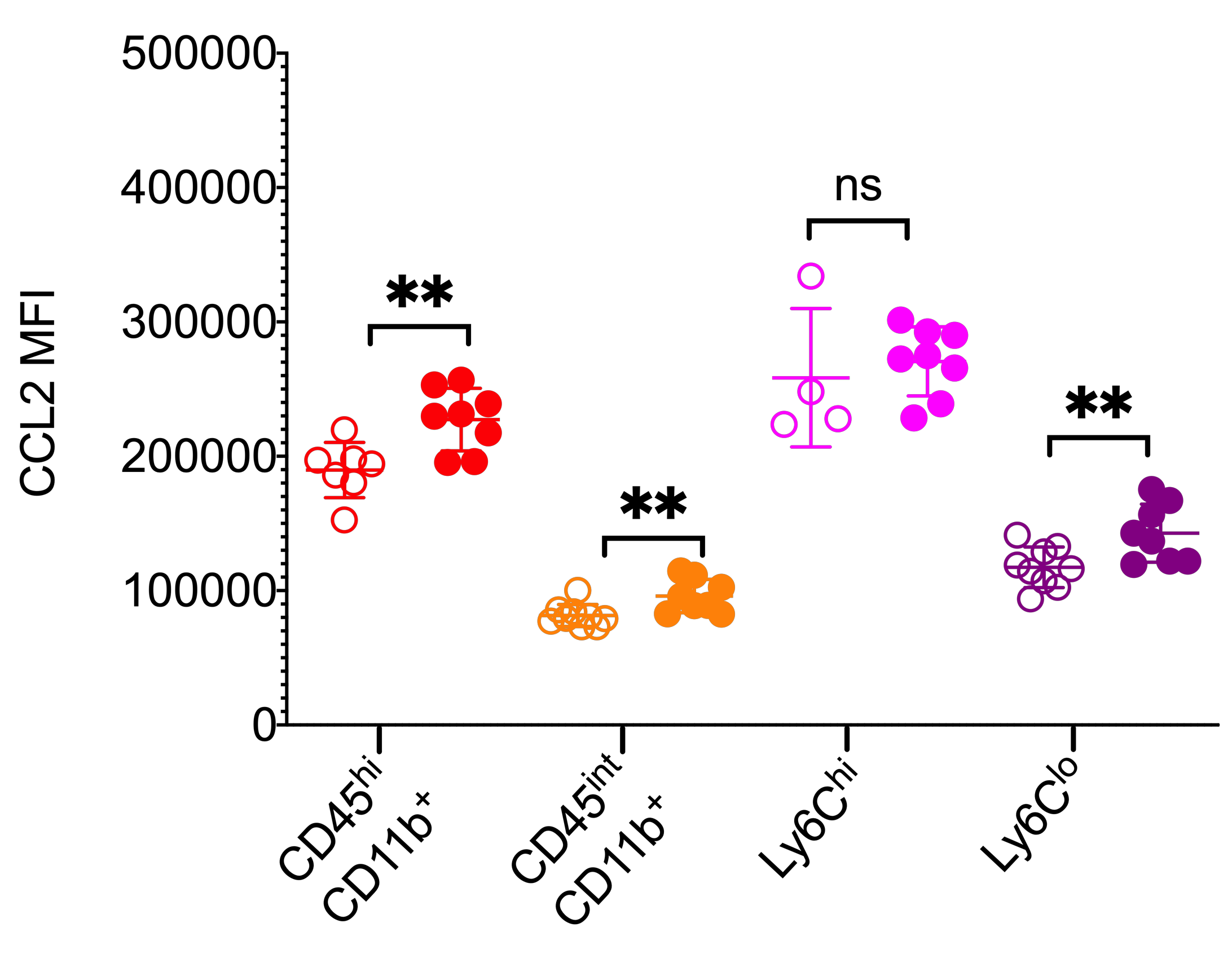

Supplement: S3 Fig — CCL2-RFP mice were injected with PBS as a control or infected with PRU strain T. gondii, and brains were harvested at 15 DPI. Immune cells from the brain homogenates were analyzed by flow cytometry, and the mean fluorescence intensity (MFI) of CCL2-RFP in CCL2-RFP+ cells from PBS-injected (open circles) or T. gondii-infected (closed circles) mice was determined. n = 4–9 mice per group from three experiments. Statistical significance was determined by randomized block ANOVA. **p<0.01, ns: not significant. (TIFF) [file ppat.1011710.s003.tiff]

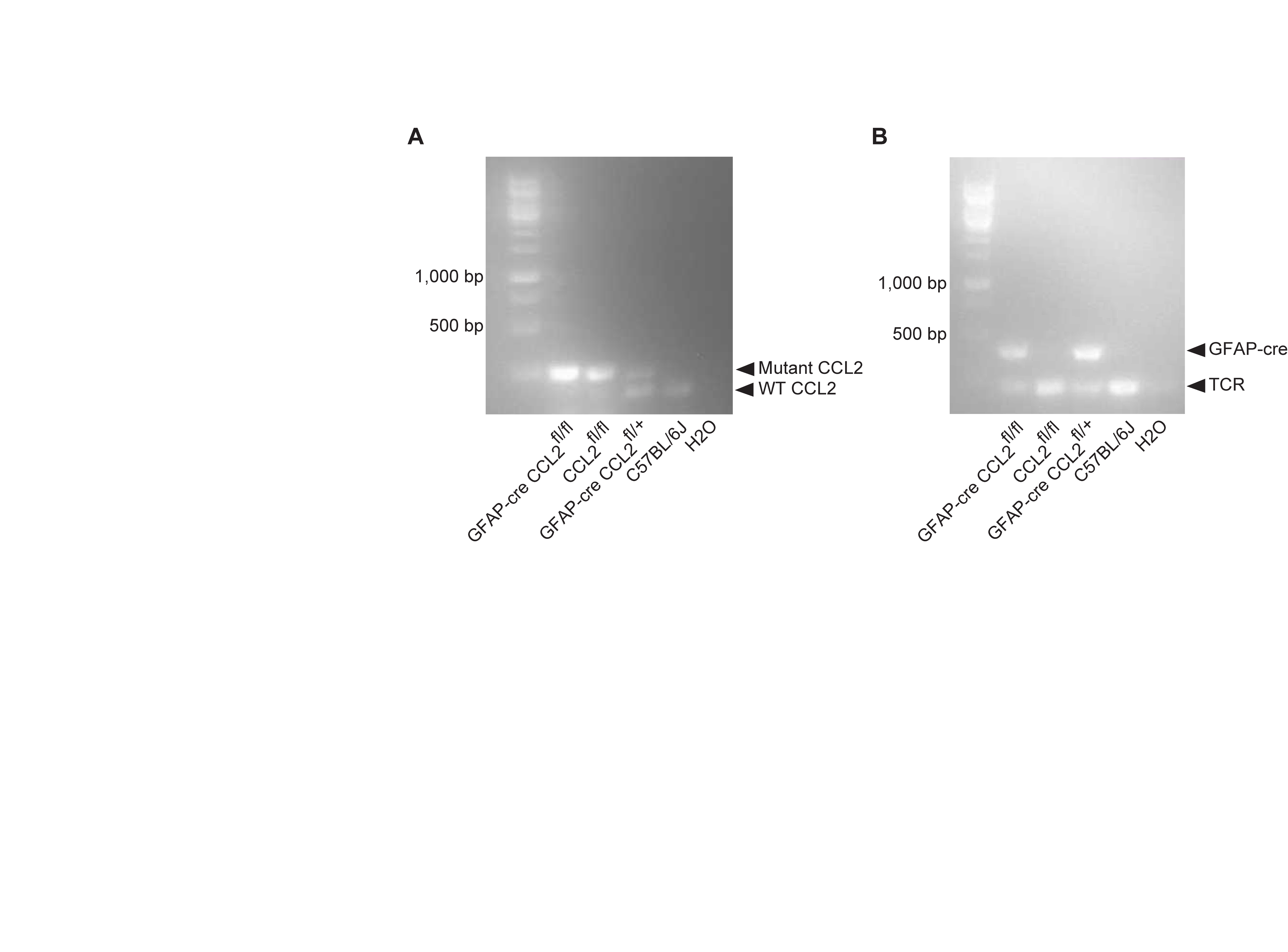

Supplement: S4 Fig — (A) Gel from PCR of genomic DNA isolated from ear punches showing the presence of floxed ccl2 (top band labeled “Mutant CCL2”) in GFAP-Cre CCL2fl/fl, CCL2fl/fl, and GFAP-Cre CCL2fl/+ mice but not in C57BL/6 mouse. Endogenous ccl2 locus without loxP sequences (bottom band) is detected in C57BL/6 mice and GFAP-Cre CCL2fl/+mice, but not in GFAP-Cre CCL2fl/fl nor CCL2fl/fl mice. (B) Gel from PCR showing the presence of cre (top band) in GFAP-Cre CCL2fl/fl and GFAP-Cre CCL2fl/+ mice but not in CCL2fl/fl nor C57BL/6J wildtype mice. To control for the presence of DNA in each sample, primers to detect the T cell receptor (TCR) (bottom band) were used. (TIF) [file ppat.1011710.s004.tif]

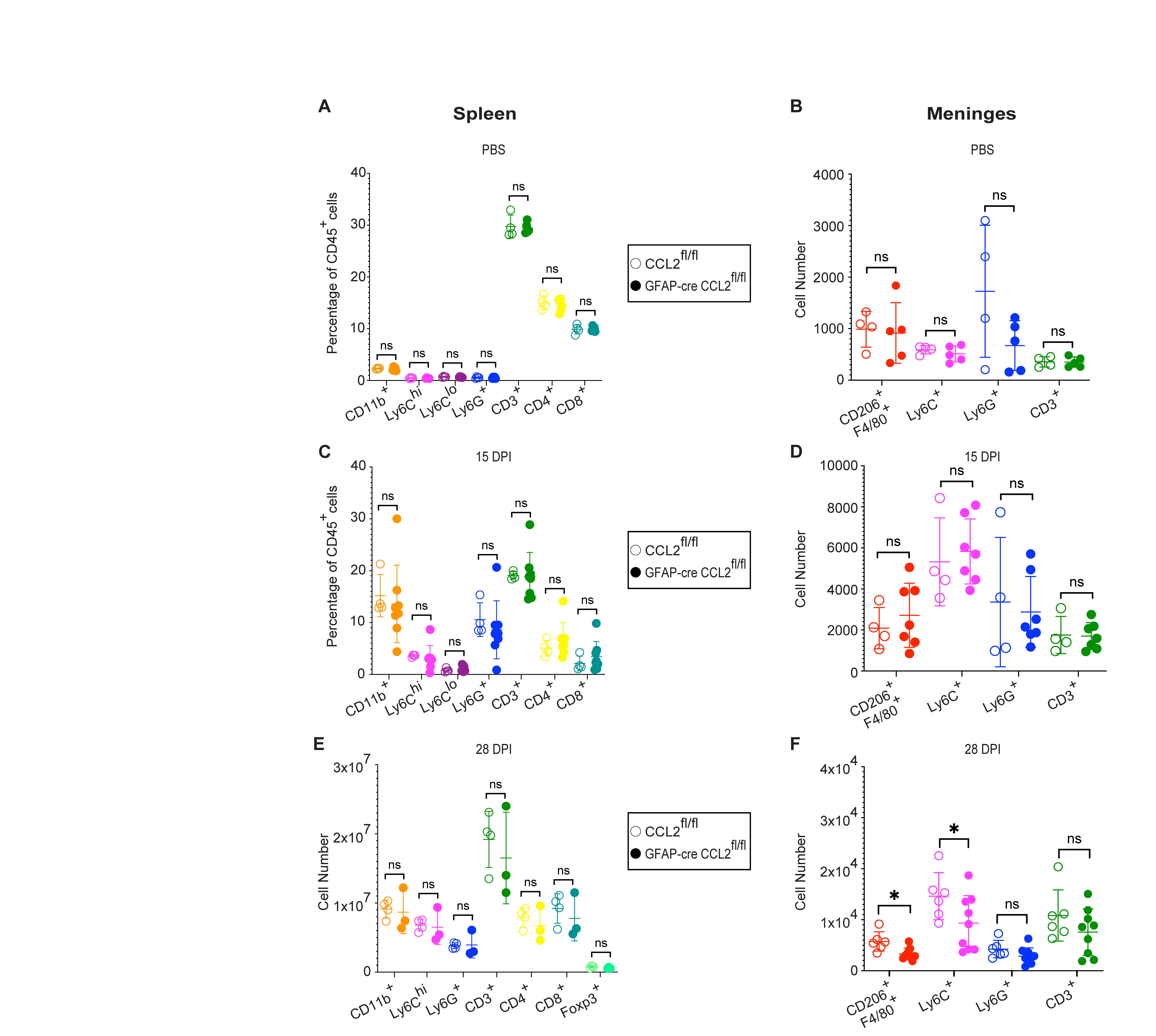

Supplement: S5 Fig — Control CCL2fl/fl (open circles) and GFAP-Cre CCL2fl/fl (closed circles) mice were injected with PBS (A and B) or infected with T. gondii (PRU 15 DPI, and ME49 28 DPI) (C-F), and spleens and meninges were harvested. (A, C, E) Frequencies of spleen CD11b+ myeloid cells, Ly6Chi monocytes, Ly6Clo monocytes, Ly6G+ neutrophils, CD3+ T cells, CD4+ T cells, and CD8+ T cells by flow cytometry. (B, D, F) Frequencies of meningeal F4/80+CD206+ macrophages, Ly6C+ monocytes, Ly6G+ neutrophils, and CD3+ T cells. In (A and B) n = 4–5 mice per group, in (C and D) n = 4–7 mice per group, in (E) n = 3–4 mice per group, and in (F) n = 6–8 mice per group from at least two independent experiments. Statistical significance was determined by a randomized block ANOVA. *p<0.05, ns: not significant. (TIF) [file ppat.1011710.s005.tif]

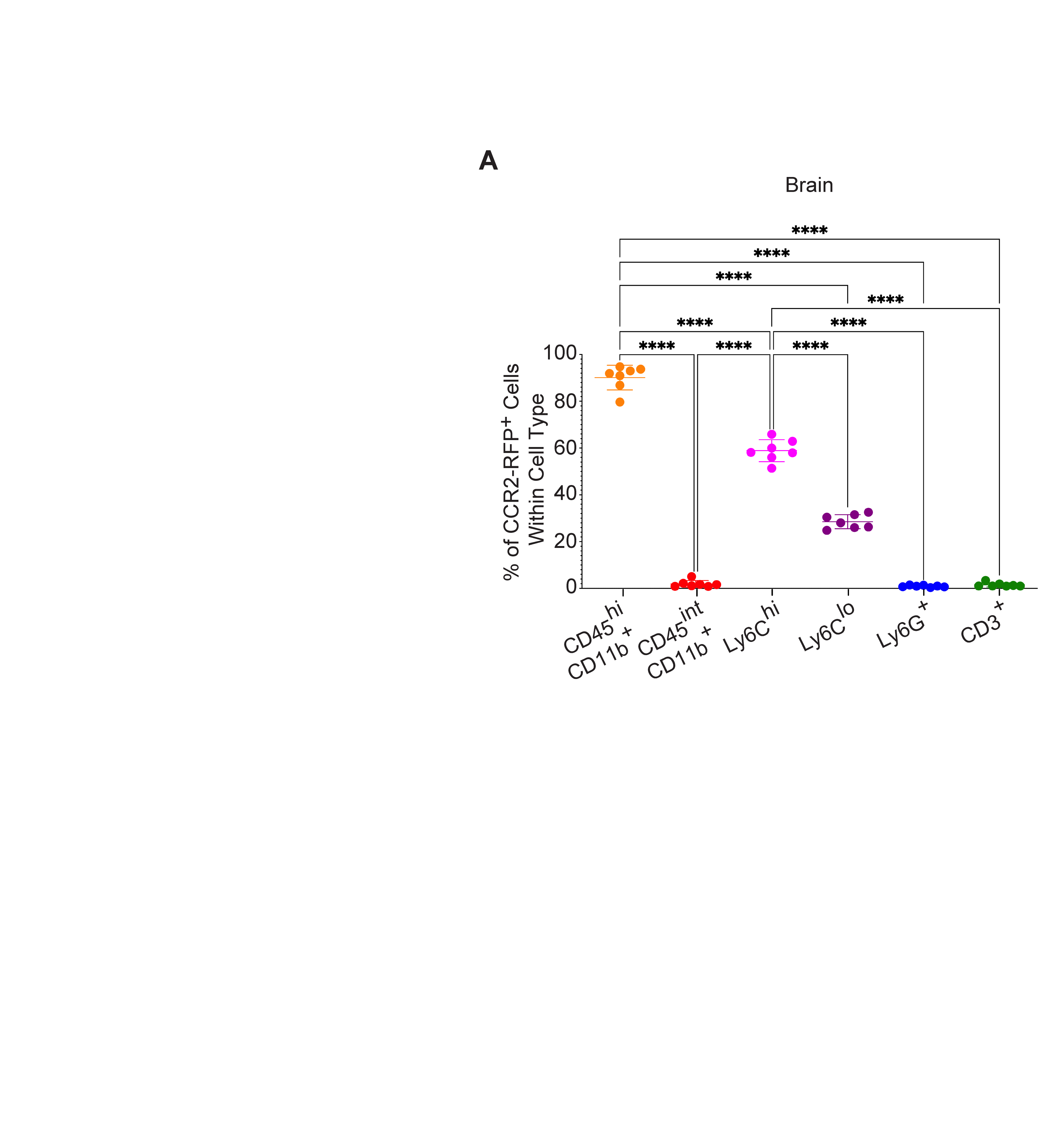

Supplement: S6 Fig — CCR2RFP/+ mice were injected i.p. with 200 T. gondii (PRU strain), and brains were harvested at 15 DPI for flow cytometry of single cells. The percent of CCR2-RFP+ cells from each immune cell population is plotted. n = 7 mice per group. Statistical significance was determined by a one-way ANOVA. ****p<0.0001. (TIF) [file ppat.1011710.s006.tif]

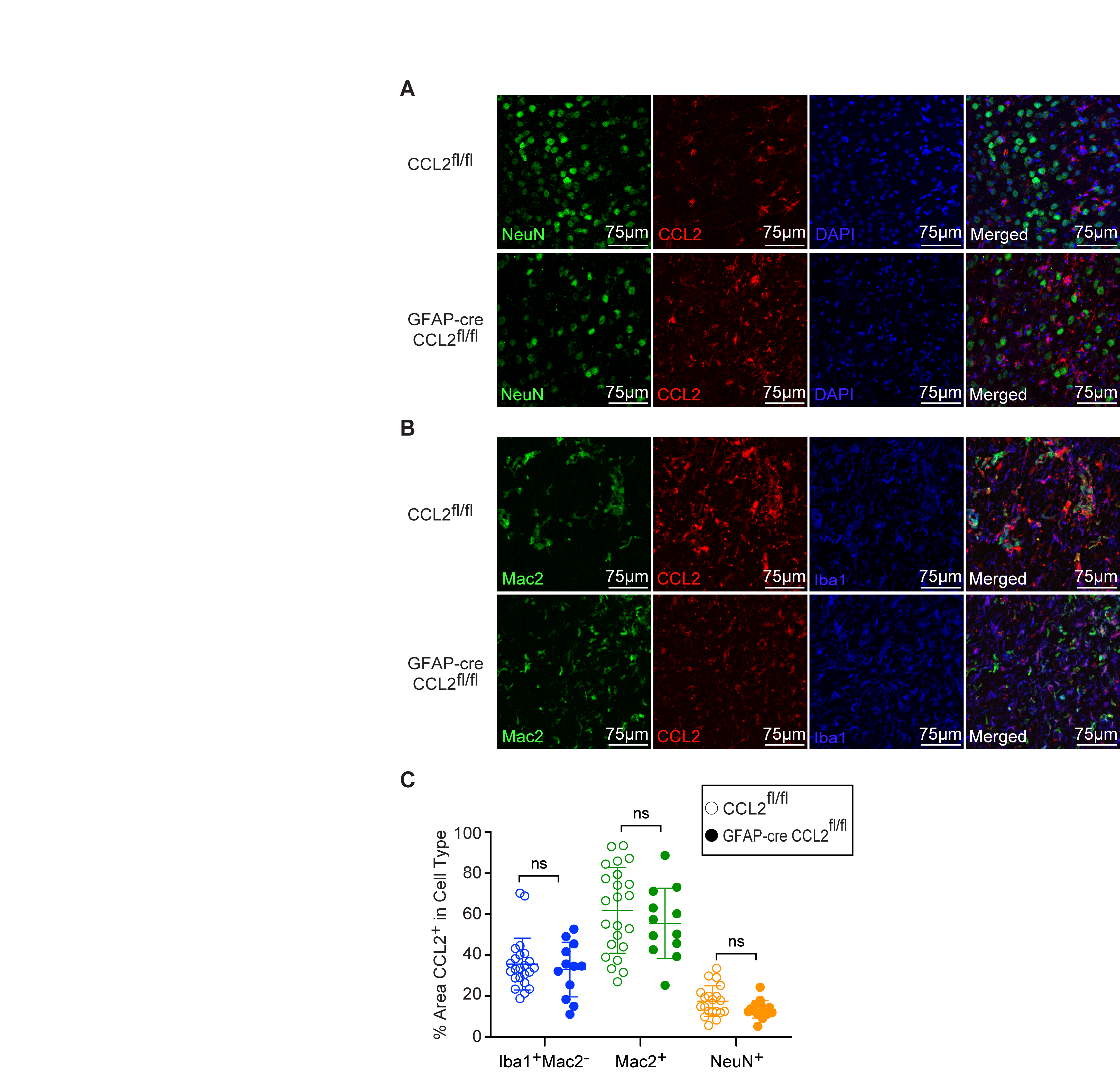

Supplement: S7 Fig — CCL2fl/fl and GFAP-cre CCL2fl/fl mice were infected with T. gondii (PRU strain) and the brains were harvested and stained with antibodies for analysis at 28 DPI. (A) Representative confocal microscopy of NeuN+ neurons (green), CCL2-RFP (red), and DAPI (blue). (B) Representative confocal microscopy of Mac2+ myeloid cells, (green), CCL2-RFP (red), and Iba1+ myeloid cells (blue). (C) Percent area of CCL2-RFP signal within each cell type. n = 12–23 FOV from 5–7 mice per group from 2 experiments. Statistical significance was calculated using Student’s t-test. ns, not significant. (TIF) [file ppat.1011710.s007.tif]

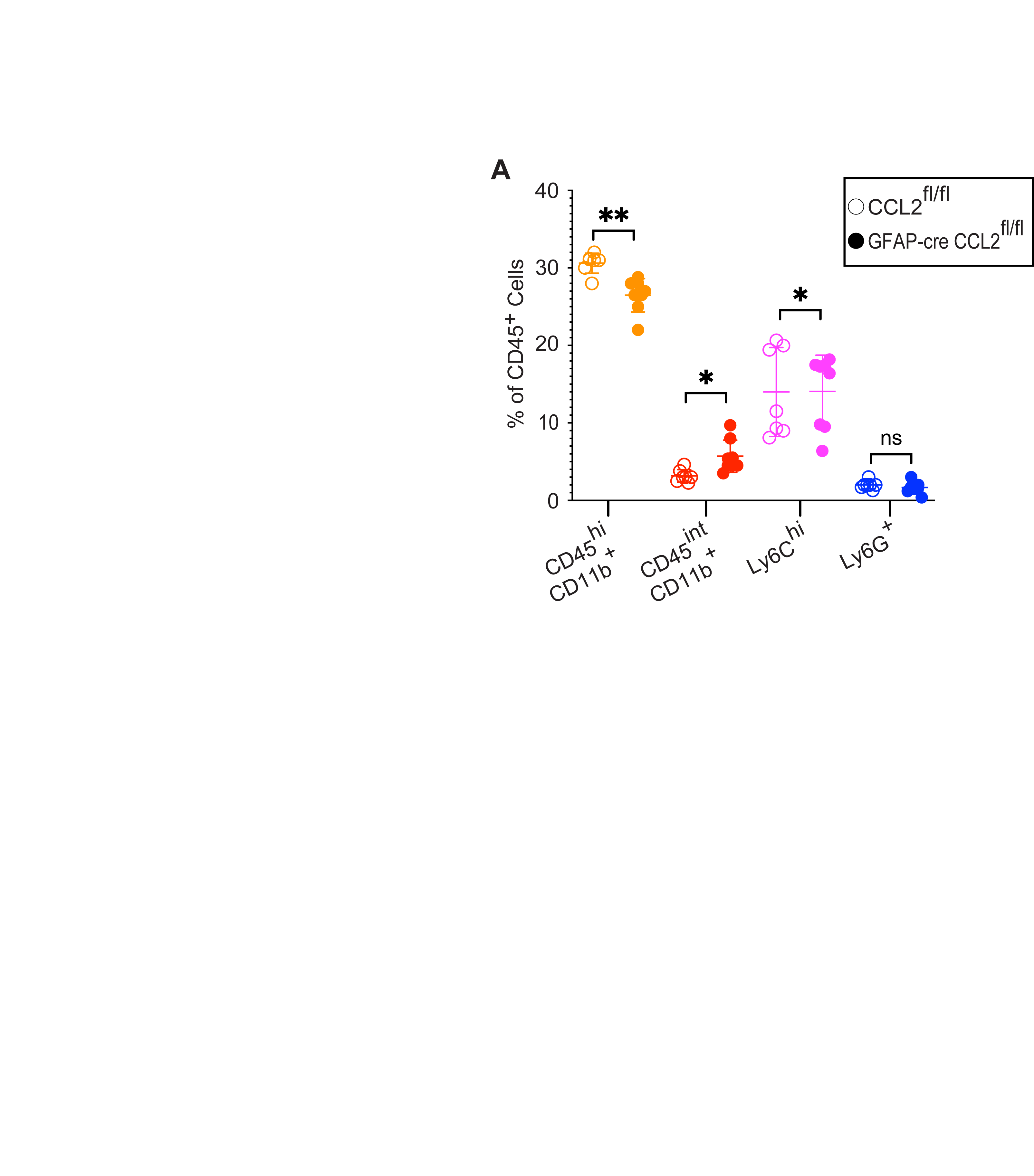

Supplement: S8 Fig — CCL2fl/fl or GFAP-cre CCL2fl/fl mice were infected with T. gondii (ME49 strain), and the brains were harvested at 28 DPI. The frequencies of myeloid immune cells in the brain were determined by flow cytometry. n = 7–8 mice per group from two experiments. Statistical significance was determined by randomized block ANOVA. *p<0.05, **p<0.01, ns, not significant. (TIF) [file ppat.1011710.s008.tif]

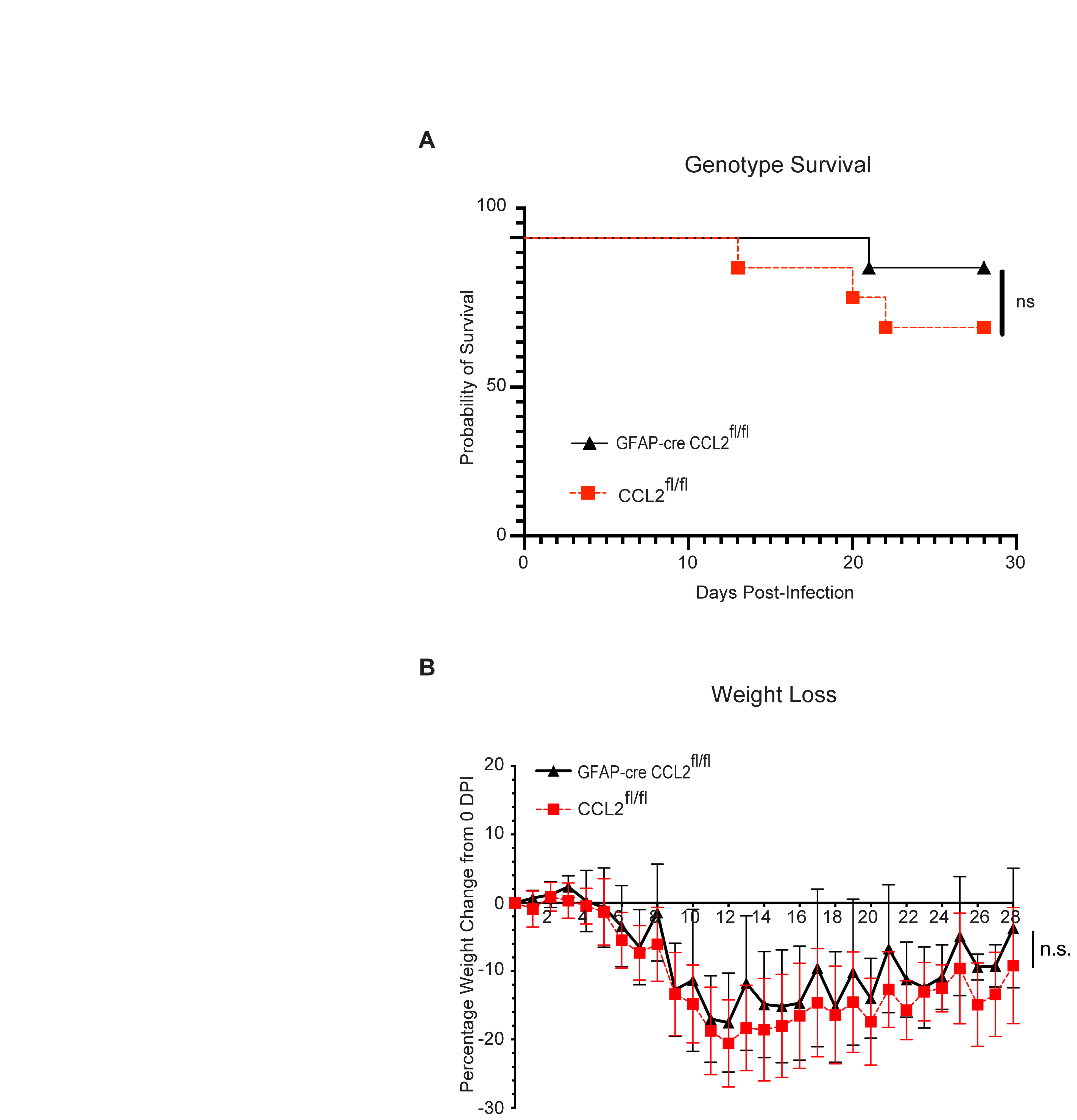

Supplement: S9 Fig — CCL2fl/fl and GFAP-cre CCL2fl/fl mice were infected with T. gondii (PRU strain) and monitored for 28 DPI. (A) Gehan-Breslow-Wilcoxon survival curves were generated for CCL2fl/fl (red) and GFAP-Cre CCL2fl/fl (black) mice. n = 10 mice per group from two experiments. (B) Weight loss curves were generated for CCL2fl/fl (red) and GFAP-Cre CCL2fl/fl (black) mice. n = 10 mice per group from three experiments. Statistical significance between the slopes of the curves were measured between 0 and 7 DPI, 7 and 14 DPI, 14 and 21 DPI, and 21 to 28 DPI using linear regression. ns, not significant. (TIF) [file ppat.1011710.s009.tif]
